# Integrated strategies for enhanced drought tolerance in tomato: from molecular mechanisms to field applications

**DOI:** 10.1093/hr/uhag227

**Published:** 2026-06-05

**Authors:** Li Ma, Huiru Wang, Bin Zhang, Yanan Guo, Siying Ou, Yue Tian, Meng Guo

**Affiliations:** School of Enology and Horticulture, Ningxia University, Yinchuan 750021, China; School of Civil and Hydraulic Engineering, Ningxia University, Yinchuan 750021, China; School of Enology and Horticulture, Ningxia University, Yinchuan 750021, China; School of Enology and Horticulture, Ningxia University, Yinchuan 750021, China; School of Enology and Horticulture, Ningxia University, Yinchuan 750021, China; School of Enology and Horticulture, Ningxia University, Yinchuan 750021, China; School of Enology and Horticulture, Ningxia University, Yinchuan 750021, China; Key Laboratory of Modern Molecular Breeding for Dominant and Special Crops in Ningxia, Ningxia University, Yinchuan 750021, China; Ningxia Modern Facility Horticulture Engineering Technology Research Center, Ningxia University, Yinchuan 750021, China; Ningxia Facility Horticulture (Ningxia University) Technology Innovation Center, Ningxia University, Yinchuan 750021, China

## Abstract

Drought stress is a major abiotic constraint on agricultural production, and cultivated tomato varieties are generally sensitive to water deficit. This review systematically summarizes the physiological, biochemical, and molecular mechanisms underlying tomato drought responses. It also integrates recent high-impact insights to advance our understanding of this topic. It then surveys multidimensional improvement strategies. These include biostimulants, chemical regulators, nanomaterials, grafting, water management, and genetic engineering approaches such as transgenesis and CRISPR/Cas9-based genome editing. Current research faces bottlenecks in translating laboratory findings to field applications, particularly due to the inherent trade-off between drought tolerance and fruit yield and quality. To address this challenge, we propose a framework that integrates several key components: precision editing tools (e.g. base editing and promoter editing), tissue-specific or stress-inducible expression systems, and parallel phenotyping of yield components and fruit quality traits under both well-watered and drought conditions. This framework is designed to balance stress resilience with productivity. Moving forward, a systematic multidisciplinary approach is required to overcome this trade-off, enabling water-efficient, stable-yielding, and high-quality sustainable tomato production. This review provides an integrative perspective and forward-looking guidance for future research and practical applications.

## Introduction

Drought poses a significant threat to human societies, ecosystems, and agricultural systems worldwide [1, 2]. Anthropogenic climate change has further exacerbated the frequency and severity of drought events. Over the past three decades, ~77.6% of the Earth’s land area has experienced arid conditions. Crop yield losses solely attributable to drought annually exceed the combined losses caused by all pathogens [2]. Tomato (*Solanum lycopersicum* L.) is a major vegetable crop cultivated globally. During domestication, tomato has lost drought tolerance and now requires substantial water. As a result, its yield and quality are highly susceptible to drought stress. Notably, rising global temperatures mean that heat stress frequently co-occurs with drought. These compound stress conditions are more detrimental than either stress alone. At the molecular level, drought and heat responses share key regulatory hubs. These include the transcription factor (TF) DEHYDRATION-RESPONSIVE ELEMENT BINDING PROTEIN 2A (DREB2A), reactive oxygen species (ROS) and calcium (Ca^2+^) signaling, and protective proteins such as heat shock proteins (HSPs). These shared components provide a mechanistic basis for cross-protection [3]. Consistently, several strategies have demonstrated efficacy against combined drought and heat stress. Examples include arbuscular mycorrhizal symbiosis and melatonin (MT) application [4, 5]. This highlights their potential for climate-resilient tomato production.

Tomato responses to drought stress include physiological, morphological, and molecular adaptations. Drought inhibits tomato photosynthesis by reducing the net photosynthetic rate, thereby restricting plant growth [6]. This reduction primarily arises from two sources: stomatal limitations (due to stomatal closure and decreased CO_2_ availability) and nonstomatal limitations (decreased photosynthetic activity in mesophyll tissues) [7, 8]. Reduced photosynthetic rates further aggravate ROS accumulation and lipid peroxidation, ultimately causing membrane damage. Under drought stress, drought-tolerant tomato varieties maintain higher root water content and a greater root-to-shoot biomass ratio than drought-sensitive varieties. This is likely due to hormonal balance [6]. Alterations in stomatal density and distribution also enhance tomato drought tolerance. These physiological and morphological adjustments depend on genomic and genetic changes. Gene expression studies have identified numerous drought-responsive genes. These provide valuable targets for breeding drought-tolerant tomato varieties.

Significant progress has recently been made in elucidating tomato drought response mechanisms. This provides a critical foundation for advancing productive breeding programs and developing innovative integrated drought-tolerance strategies. This review synthesizes traditional agronomic practices, emerging biotechnology and nanotechnology approaches, omics-driven gene discovery, and cross-disciplinary methods. Together, these approaches bridge molecular mechanisms with field applications. We propose that multidimensional synergistic enhancement represents a critical pathway to overcome current limitations in tomato drought tolerance.

## Drought response mechanisms of tomato

Drought resistance in plants involves perceiving water deficits and activating multifaceted adaptive strategies. These strategies fall into three categories: drought escape, drought avoidance, and drought tolerance [9]. Drought escape enables plants to complete their life cycle before severe stress occurs. Drought avoidance maintains tissue water potential through morphological adjustments. Drought tolerance allows plants to sustain physiological functions under low tissue water potential via osmotic and protective mechanisms. Tomato responses to water scarcity involve coordinated morphological, physiological, and molecular changes. These changes reflect a trade-off between growth, reproduction, and survival (Fig. 1). This trade-off operates in an integrated and context-dependent manner.

**Figure 1:**
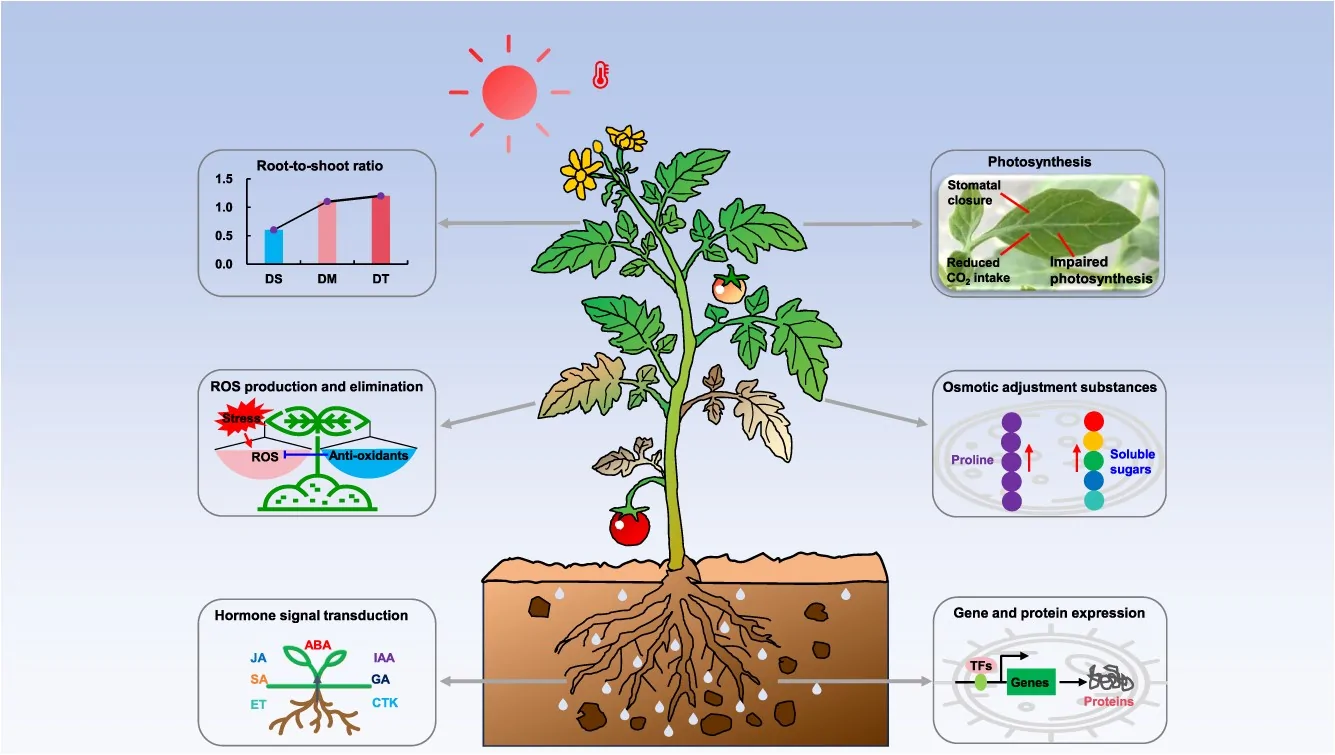
Drought stress response in tomato plants. The response of tomato to drought stress encompasses morphological, physiological, and molecular changes. Morphological changes mainly include leaf water loss, wilting, and yellowing after drought stress, with significant differences in root-to-shoot ratios observed among tomato varieties with different drought tolerances. Physiological changes primarily involve ROS scavenging, hormone signaling, photosynthesis, and osmotic adjustment. Molecular changes mainly refer to alterations in the expression levels of stress-induced genes and proteins within tomato plants. The upward arrows indicate the accumulation of osmotic solutes. DS, drought-sensitive cultivar; DM, moderately drought-tolerant cultivar; DT, drought-tolerant cultivar; ROS, reactive oxygen species; JA, jasmonic acid; SA, salicylic acid; ET, ethylene; ABA, abscisic acid; IAA, indole-3-acetic acid; GA, gibberellin; CTK, cytokinin; TFs, transcription factors.

### Regulatory network of tomato flower development under drought stress

Tomato is susceptible to drought at all growth stages, particularly during flowering. Accelerating flowering serves as a drought escape strategy [9]. A well-established mechanism involves the protein kinase OPEN STOMATA 1 (SlOST1), activated by drought. SlOST1 stabilizes and phosphorylates the TF VASCULAR PLANT ONE-ZINC FINGER 1 (SlVOZ1), promoting its nuclear entry. SlVOZ1 then activates transcription of the flowering gene *SINGLE FLOWER TRUSS* (*SFT*), inducing flowering [10]. This provides a clear molecular pathway from stress perception to adaptive flowering.

Conversely, tomato also regulates floral organ abscission in response to drought, often conflicting with the escape strategy. In the ethylene (ET) pathway, drought downregulates the *demethylase alpha-ketoglutarate-dependent dioxygenase B (AlkB) homolog 9B* (*SlALKBH9B*) in abscission zones. This increases the methylation of ET excess producer 1 (SlETO1), impairing its function. As a result, 1-aminocyclopropane-1-carboxylic acid synthase 3 (SlACS3) and SlACS8 accumulate. Enhanced ET biosynthesis then triggers flower shedding [11]. In the auxin pathway, drought activates a calcium module composed of calcineurin B-like 11 (SlCBL11)-CBL-interacting protein kinase 10 (SlCIPK10). This module phosphorylates like heterochromatin protein 1b (SlLHP1b), which subsequently suppresses the stamen-specific auxin biosynthesis gene *YUCCAs* (*SlYUCs*). This disrupts the auxin gradient and contributes to floral abscission [12]. The peptide hormone phytosulfokine (PSK) also accelerates abscission by inducing cell wall hydrolases [13]. Together, these findings reveal a complex regulatory network. However, the mechanism determining whether tomato accelerates flowering or induces abscission under specific stress conditions remains unclear.

### Morphological adaptation–driven drought avoidance strategies

Maintaining high tissue water potential through morphological adaptations is a key drought-avoidance strategy. A prominent adaptation is an increased root-to-shoot ratio, as observed in drought-tolerant landraces like ‘Ciettaicale’. This adaptation enhances water uptake and sustains plant water status [6, 14]. The shift represents a strategic redistribution of carbon from source to sink tissues, prioritizing root development. Additionally, a key anatomical adaptation contributing to drought tolerance in tomato is the presence of a suberized exodermis. In tomato, suberin is deposited specifically in the exodermis rather than the endodermis. There, it functions as an apoplastic diffusion barrier. The TF SlMYB92 and ALIPHATIC SUBERIN FERULOYL TRANSFERASE (SlASFT) are essential for exodermal suberin deposition. Loss-of-function mutants exhibit impaired drought tolerance due to disrupted water relations [15].

Beyond these structural barriers, roots also employ directional growth strategies to forage for water. Tomato contains three hydrotropism-associated loci (*MIZ1-like*), among which *SlMIZ1-1* is indispensable for root hydrotropism. CRISPR/Cas9-mediated triple knockout of *SlMIZ1-1*, *SlMIZ1-2*, and *SlMIZ1-3* impairs hydrotropic bending and reduces lateral root development. This leads to a diminished capacity to recover from drought episodes. Drought significantly induces the expression of all three *SlMIZ1* genes. *SlMIZ1-1* complements the *Arabidopsis miz1* mutant, restoring root hydrotropism, waviness, and Ca^2+^ signaling. These findings establish *SlMIZ1-1* as a key regulator of root water foraging and postdrought recovery [16].

Improving water use efficiency (WUE) is also essential, governed by stomatal conductance, transpiration, and photosynthetic rates [17]. Maintaining a low vapor pressure deficit (VPD) optimizes WUE by reducing transpirational demand and improving leaf water potential. Gibberellin (GA) is a well-established hormonal regulator. Drought reduces bioactive GA levels, which promotes stomatal closure and represses leaf growth. This reduction in transpiration enables rapid and sustained drought avoidance [18]. Additional evidence from other pathways supports this strategy. For instance, knocking out the SA-related gene *DOWNY MILDEW RESISTANCE 6-1* (*SlDMR6-1*) decreases stomatal conductance and thereby reduces transpiration. This further enhances drought avoidance [19].

Under mild drought, initial stomatal closure can excessively suppress electron transport chain. This increases ROS production and lipid peroxidation. As stress intensifies, damage to chloroplast membranes and photosystem II (PSII) further reduces net photosynthetic rate and light-use efficiency [6]. However, the ability to sustain photosynthetic function under stress depends on genotype [6, 17]. Tolerant tomato genotypes better manage these limitations, sustaining photosynthesis. This trait can be achieved by grafting commercial scions onto drought-tolerant rootstocks [20].

### Dual defense mechanisms of tomato drought tolerance

Under drought, ROS cause oxidative damage. However, they also act as key signaling molecules that activate defense responses. This duality is critical to understanding plant adaptation [21, 22]. At moderate levels, ROS function as secondary messengers. For instance, hydrogen peroxide (H_2_O_2_) mediates ABA-induced stomatal closure and activates antioxidant defenses in tomato [23, 24]. Enhanced drought tolerance depends on maintaining ROS homeostasis via a robust antioxidant system. This includes upregulated antioxidant enzymes and nonenzymatic antioxidants, such as lycopene, phenols, and flavonoids. These compounds may increase under certain deficit irrigation (DI) regimes without compromising fruit quality [25]. Hormonal signals often coordinate this response. For example, the *ABA-responsive element binding protein 1* (*SlAREB1*) enhances drought tolerance by improving both ROS scavenging and photosynthetic efficiency [26]. The balance between ROS signaling and oxidative damage is tightly regulated by stress timing, intensity, and duration. Mild stress can induce acclimation. In contrast, prolonged or severe stress causes excessive ROS accumulation, which surpasses the scavenging capacity and leads to cellular damage [27]. Although external SA can further enhance antioxidant capacity, whether this effect is direct or indirect remains unclear [28].

Recent studies have uncovered novel regulatory nodes governing ROS homeostasis in guard cells. For instance, the heat shock protein DnaJ1 interacts with the flavanone 3-hydroxylase-like protein SlF3HL. This interaction synergistically enhances the activity of antioxidant enzymes, thereby reducing H_2_O_2_ and superoxide anion radicals (O_2_^·–^) accumulation under drought stress [29]. Additionally, the Rho GTPase SlROP9 negatively regulates respiratory burst oxidase homolog (RBOH)–dependent ROS production in guard cells. *slrop9* null mutants therefore show constitutively higher ROS levels, enhanced stomatal closure, and improved WUE without yield penalty [30]. Sustaining ROS homeostasis and antioxidant capacity is directly relevant to yield stability under drought. Genotypes or treatments with higher antioxidant enzyme activity typically experience less drought-induced flower and fruit abscission. This results in consistent yield and maintained fruit quality, including lycopene and ascorbic acid content [28, 31].

To cope with cellular dehydration, plants accumulate compatible solutes in a process called osmotic adjustment. Proline is a multifunctional osmoprotectant in tomato, lowering osmotic potential, stabilizing membranes and proteins, reinforcing cell walls, and scavenging ROS. However, proline accumulation under drought is not consistently associated with tolerance. In a study of eight wild tomato accessions, two drought-tolerant lines (ECU689 and ECU1009) showed no significant proline increase under stress. In contrast, more sensitive accessions accumulated substantial proline [32]. This indicates that proline accumulation is not a prerequisite for drought tolerance in tomato, highlighting divergence in metabolic adaptation among genetic backgrounds. Thus, proline accumulation alone is an unreliable indicator of drought tolerance. Its functional role likely depends on stress conditions and genetic context.

Soluble sugars also contribute to osmotic balance, but their role in drought-stressed tomato is context-dependent. Several studies show that enhanced sugar accumulation correlates with improved tolerance. For example, overexpression of dehydrin gene *tas14* promotes sugar and potassium accumulation, reducing osmotic potential [33]. The calcium-dependent protein kinase 27 (CPK27) phosphorylates the tonoplast sugar transporter 2 (TST2) in a Ca^2+^/ABA-dependent manner. Disruption of this pathway reduces tolerance [34]. Conversely, ABA-deficient mutants constitutively accumulate higher sugar levels, partially compensating for stomatal defects [35]. However, sugar accumulation does not always confer tolerance. The sensitive cultivar ‘Moneymaker’ accumulates more soluble sugars under stress than the tolerant ‘Ciettaicale’. However, this reflects disrupted source–sink relationships rather than enhanced resilience [14]. Thus, the significance of sugar accumulation depends on genetic background, stress conditions, and whether it represents coordinated adaptation or carbon sink limitation.

Polyamines (PAs) include putrescine (Put), spermidine (Spd), and spermine (Spm). PAs contribute to tomato drought tolerance through various physiological and molecular mechanisms. Under drought stress, tomato leaves exhibit increased levels of Spd and Spm, particularly in their conjugated and bound forms, whereas Put levels decline. These metabolic changes are accompanied by differential regulation of biosynthesis genes, such as *arginase 1* (*SlARG1*), *Spd synthase 1* (*SlSPDS1*), and *S-adenosylmethionine decarboxylase 3* (*SlSAMDc3*). The catabolic gene *copper-dependent amine oxidase 4-like* (*SlCuAO4-like*) is also differentially regulated [36]. Genetic evidence confirms the protective roles of PAs. CRISPR/Cas9 knockout of *SlPAO3* (encoding a PA oxidase) elevates thermospermine (T-Spm) levels. This reduces xylem hydraulic conductivity and embolism vulnerability, thereby enhancing drought tolerance without growth penalty [37]. Similarly, overexpression of *SlSPDS1/4* boosts ROS scavenging and mitigates wilting under drought stress [38]. In grafted tomato fruits under water deficit, higher free Spd correlates with increased ADC and SPDS activities, elevated SOD and CAT levels, and reduced H_2_O_2_ and MDA [39]. Collectively, Spd serves as a key protective PA in tomato drought responses. In contrast, the role of Put appears limited. Its metabolic pathways thus offer promising targets for genetic improvement.

### ABA signaling and hormonal crosstalk

Phytohormones, particularly ABA, act as master regulators of drought responses. ABA is rapidly synthesized upon drought perception, modulating water balance and promoting stomatal closure to enhance tolerance [40]. The signaling component SlOST1 regulates guard cell activity, and its mutation reduces stomatal sensitivity to drought [10]. Comparative studies between high-ABA plants and ABA-deficient mutants confirm that ABA primarily delays dehydration by reducing water loss, reinforcing drought tolerance [41]. This central role of ABA is well established in plant drought physiology.

However, interactions between ABA and other hormones are complex and context-dependent. A characterized antagonistic mechanism shows that ABA induces IAA oxidation and deactivation via DIOXYGENASE FOR AUXIN OXIDATION2 (SlDAO2), thereby inhibiting hypocotyl elongation. Notably, this effect occurs under exogenous ABA treatment, rather than under drought itself [42]. In contrast, ABA-deficient mutants indicate that IAA partially compensates for ABA, sustaining root growth under drought [43]. Further complexity arises from SlIAA9, an Aux/IAA family member. *SlIAA9* is transcriptionally induced by ABA and negatively regulates drought tolerance by affecting ROS scavenging [44]. These findings suggest that ABA-IAA interplay varies with tissue type, stress severity, developmental stage, and the molecular nodes involved. This variation differentially modulates root and shoot growth. Nevertheless, its net effect on water productivity under field conditions remains to be validated. Such validation requires integrated physiological, molecular, and agronomic approaches across diverse genetic backgrounds and environments.

ABA and JA interact synergistically via the ET-responsive factor D2 (ERF.D2), a crucial regulatory node. ABA promotes the degradation of ERF.D2. This degradation alleviates ERF.D2-mediated suppression of JA biosynthesis genes and activates JA-dependent defensive responses, thereby enhancing drought tolerance [45]. SA reduces drought-induced stress through modulation of ABA concentrations and stomatal aperture [28], whereas brassinosteroids (BRs) ameliorate drought damage by inducing ABA accumulation or by increasing antioxidant enzyme activity [46]. Strigolactones (SLs) share a common biosynthetic precursor with ABA. They function through organ-specific interactions to establish systemic drought signaling, independent of root-derived ABA transport [47]. This indicates redundancy, as well as specialization, within these hormonal signaling networks.

ABA also exhibits functional synergy with GA. Under drought, ABA-dependent and ABA-independent pathways suppress GA activity, leading to the accumulation of aspartic acid–glutamic acid–leucine–leucine–alanine (DELLA) proteins [18]. These proteins reinforce ABA signaling. They rapidly induce stomatal closure while inhibiting leaf expansion and reducing transpiration area. This interaction is well recognized as an example of hormonal crosstalk coordinating both rapid and sustained adaptive responses.

Beyond the ABA-GA interplay, the ABA-dependent and ABA-independent pathways intersect at multiple levels. The core ABA-dependent module comprises PYR/PYL/RCAR receptors, SlOST1/sucrose nonfermenting-1-related protein kinase 2 (SnRK2) kinases, and AREB/AREB factor (ABF) TFs such as SlAREB1. Overexpression of *SlAREB1* enhances drought tolerance primarily by up-regulating genes encoding oxidative stress–related proteins and molecular chaperones [26]. In parallel, ABA-independent pathways are mediated by dehydration-responsive element-binding (DREB) TFs. Notably, SlMAPK3 is transcriptionally induced by drought, salt, and exogenous ABA [48, 49]. SlMAPK3 positively regulates drought tolerance by promoting osmolyte accumulation, enhancing antioxidant enzyme activities, and reducing oxidative membrane damage [48, 49]. A key mechanism of pathway convergence occurs at the transcriptional level. In *slmapk3* mutants, the expression of both the antioxidant gene *SlGST* and the ABA-independent TF *SlDREB* is significantly down-regulated under drought stress [49].

Furthermore, physical interaction between ABA-dependent and ABA-independent TFs provides a direct integration mechanism. SlMYB78-like, which functions through the ABA pathway, directly binds the promoter of the ABA degradation gene *SlCYP707A2* and represses its transcription. This repression maintains higher endogenous ABA levels under stress. SlMYB78-like physically interacts with SlDREB3, forming a transcriptional complex that potentially integrates regulatory inputs from both pathways to fine-tune downstream gene expression [50].

A newly identified ABA signaling cascade involves the miR398b-CSD1 module. The TF SlABF2, activated by SlSnRK2.6, directly binds to the ABA-responsive element (ABRE) motif in the sly-miR398b promoter and activates its transcription. The resulting sly-miR398b represses *Cu/Zn-SOD1* (*SlCSD1*), leading to O_2_^·–^ accumulation in guard cells and promoting stomatal closure. This mechanism operates independently of H_2_O_2_ and provides a fine-tuning mechanism for ABA-mediated ROS signaling [51]. Together, this multilayered crosstalk enables tomato plants to mount a coordinated defense against drought. Elucidating the hierarchy of these convergent interactions remains an important area for future investigation.

The interactions between ABA and both cytokinin (CTK) and ET display greater variability. CTKs do not interact directly with ABA. Instead, they influence baseline transpiration, creating a physiological state conducive to ABA functionality under drought stress. This represents an indirect and permissive role [52]. Under simultaneous competition and drought stress, ABA and ET levels increase synergistically. Their relative concentrations govern stomatal closure through a nonhydraulic mechanism under mild to moderate stress conditions. In contrast, a shift to hydraulic signaling occurs under severe drought [53]. This suggests that the nature of ABA-ET interactions varies according to stress intensity.

ABA mediates drought-induced rhizosheath formation in tomato, which improves water and nutrient uptake. However, the underlying mechanisms remain incompletely understood [54]. Soluble sugars in tomato leaves participate in ABA-mediated drought responses [35]. Trehalose-induced drought tolerance depends on ABA signaling, rather than on ABA metabolism itself [55], whereas hydrogen-enhanced tolerance results from modulation of ABA biosynthesis and signaling pathways [56]. Collectively, these findings underscore ABA’s central role as a regulator. It integrates diverse physiological and molecular responses to confer drought tolerance in tomato.

These results position ABA as a central node within a complex network of phytohormonal interactions. Although the core ABA signaling pathway is clearly defined, the spatiotemporal dynamics and hierarchical interactions among related pathways remain unclear, especially under fluctuating drought conditions. Many proposed crosstalk mechanisms, particularly those involving SLs, BRs, and ABA-ET interactions, remain speculative or are based on limited studies and specific genotypes.

In summary, drought response in tomato involves an integrated suite of processes: accelerated flowering, morphological reallocation of resources toward roots, stomatal closure, restricted leaf growth and transpiration, photosynthetic adjustment, ROS detoxification, osmoprotectant accumulation, and ABA-centered hormonal crosstalk. To effectively enhance drought tolerance in this crop, it is essential to comprehensively understand these interconnected pathways and to clearly distinguish universal mechanisms from context-dependent responses and speculative interactions.

### Genotype-dependent responses to agronomic and environmental interventions

Within the genotype–environment–management (G × E × M) framework, the effectiveness of agronomic practices and environmental regulation is significantly influenced by tomato genetic background. These differences manifest at multiple biological levels. Firstly, morpho-physiological traits display considerable genotypic variation. Under water deficit, drought-tolerant landraces such as ‘Ciettaicale’ increase their root-to-shoot ratio. This response is not evident in sensitive cultivars like ‘Moneymaker’ under identical conditions [6, 14]. This differential root plasticity underlies the efficacy of grafting practices, as scion performance under drought stress directly correlates with rootstock vigor [20, 57]. Secondly, hormonal sensitivity determines plant responses to microbe interactions. For example, the growth-promoting rhizobacteria (PGPR) *Bacillus megaterium* confers drought tolerance by downregulating ET biosynthesis in ET-sensitive wild-type tomato plants. However, this effect is absent in ET-insensitive *never ripe* mutants, highlighting a genetic basis for biostimulant efficacy [58, 59]. Thirdly, metabolic adjustment strategies differ among genotypes. Proline accumulation is not mandatory for drought tolerance. Tolerant wild accessions such as ECU689 and ECU1009 do not accumulate significant proline under stress, whereas sensitive ones do [32]. Similarly, sugar accumulation facilitates osmotic adjustment in certain genotypes. However, in the sensitive cultivar ‘Moneymaker’, it reflects sink limitation rather than improved resilience [14, 34]. Fourthly, responses to elevated CO_2_ (eCO_2_) are genotype-specific, influenced by endogenous ABA concentrations. In wild-type tomato, eCO_2_ enhances biomass, water status, and WUE under drought. These benefits are absent in ABA-deficient mutants. This indicates that genetic background governs the effectiveness of eCO_2_ as an environmental regulator [60, 61].

Collectively, genotype-specific characteristics, including root architecture, hormonal responsiveness, and metabolic flexibility, determine the effectiveness of agronomic and environmental interventions. Thus, a clearly defined G × E × M framework that incorporates these differences is crucial for practical field applications. Building upon this understanding of genotype-dependent responses, the following sections discuss strategies to enhance drought tolerance, highlighting how interventions can be customized to different genetic backgrounds.

## Regulator strategies for enhancing drought tolerance in tomato

Strategies for improving drought tolerance in crops include the use of biological regulators (biostimulants) and non-biological regulators. Biostimulants, such as arbuscular mycorrhizal fungi (AMF), PGPR, humic acids, seaweed extracts, and small-molecule natural compounds, enhance soil properties, water retention, nutrient absorption. They also improve root architecture, antioxidant capacity, and stress signaling pathways. Together, these improvements optimize plant physiology and enhance drought tolerance. Non-biological regulators comprise hormone analogues (e.g. MT), beneficial elements (such as silicon and selenium), and soil amendments (e.g. biochar). Additionally, green-synthesized nanoparticles (NPs) have emerged as a promising nanotechnology-based strategy to address drought stress at the soil–plant interface. This section systematically reviews the applications of biostimulants, non-biological regulators, and nanomaterials in enhancing drought tolerance in tomato.

### Biostimulants enhance tomato drought tolerance

AMF establish mutualistic associations with tomato roots, fundamentally enhancing drought tolerance through coordinated physical, physiological, and molecular adaptations (Fig. 2). An extensive extraradical hyphal network effectively extends the root system. This significantly increases the plant’s capacity to access soil water and nutrients, forming a primary basis for drought alleviation. Beyond physical extension, AMF regulate essential physiological processes. For instance, AMF colonization delays the soil moisture threshold at which transpiration inhibition occurs. This enables plants to sustain growth for longer periods under progressive drought [62]. At the molecular level, a crucial mechanism involves the modulation of aquaporins (AQPs), protein channels facilitating cellular water movement. AMF colonization elevates expression levels of specific host *AQP* genes (e.g. NOD26-like intrinsic protein *LeNIP3;1*) and fungal *AQP* genes (e.g. *RiAQPF2*). Consequently, this improves root hydraulic conductivity and water transport under drought conditions [63]. These AMF-induced enhancements in substrate hydraulic properties have been observed. However, their generalizability across varied soil types and cropping systems warrants further investigation.

**Figure 2:**
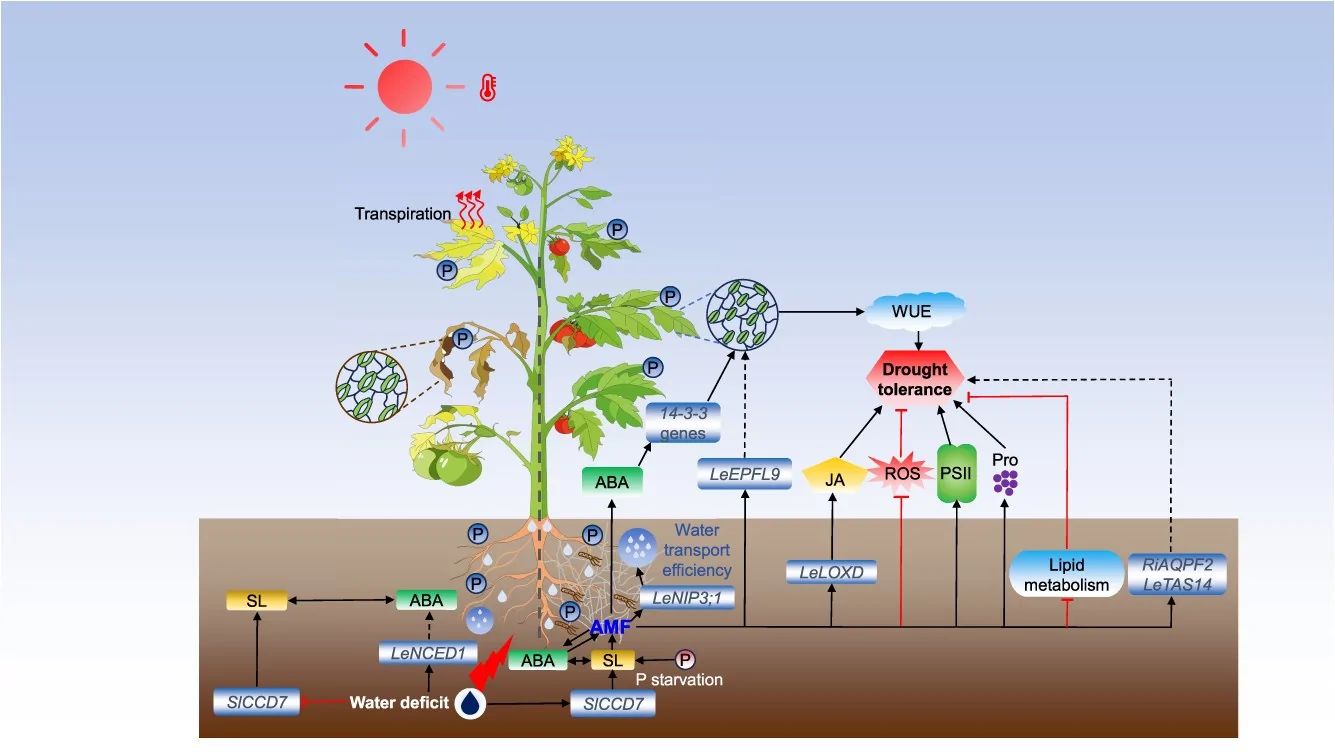
Enhancement of tomato drought tolerance by AMF inoculation. AMF enhance tomato drought tolerance through multi-level synergistic mechanisms: at the physical level, they improve water absorption and transport; at the molecular level, they regulate AQPs and hormone signaling pathways (e.g. SL, ABA, JA) to coordinately optimize stomatal movement and water balance; at the physiological level, they enhance antioxidant capacity to reduce oxidative damage, maintain higher PSII efficiency, and modulate osmoprotectants (e.g. proline) to improve cellular water retention. Furthermore, lipid exchange between AMF and the host is crucial for maintaining symbiotic functionality. These interacting mechanisms collectively form an integrated drought tolerance enhancement network. Arrows denote promotion, bars indicate inhibition, and dashed lines represent unverified pathways or associations. P, phosphorus; ABA, abscisic acid; SL, strigolactone; ROS, reactive oxygen species; JA, jasmonic acid; PSII, photosystem II; Pro, proline; WUE, water use efficiency.

The establishment and functionality of the AMF symbiosis during drought are intricately connected to the reprogramming of host hormonal signaling. SLs, secreted from roots in response to drought, function as rhizospheric signals that attract AMF hyphae. Interestingly, the symbiotic association itself actively stimulates SL biosynthesis in tomato during drought. This stimulation correlates with carotenoid cleavage dioxygenase *SlCCD7* expression. In contrast, drought alone suppresses SL biosynthesis in non-mycorrhizal plants. This indicates a symbiosis-specific adaptive response, apparently independent of phosphorus-mediated pathways [64, 65].

ABA is essential both for establishing a functional symbiotic relationship and mediating drought responses. Under drought, AMF colonization promotes host ABA biosynthesis. Elevated ABA levels subsequently induce stomatal closure by regulating downstream signaling components such as *14-3-3* genes. This action reduces transpirational water loss and enhances overall WUE [65]. While positive correlations between SL and ABA accumulation exist in mycorrhizal plants under stress, the exact nature of their crosstalk within the symbiotic context remains incompletely defined [64].

Additionally, AMF colonization upregulates JA biosynthesis genes, such as tomato *lipoxygenase D* (*LeLOXD*) under drought [63]. JA-deficient mutants display diminished AMF-derived benefits, highlighting JA’s essential role in facilitating the symbiotic enhancement of drought tolerance [66].

AMF confer broad-spectrum drought tolerance through multiple, synergistic mechanisms. They notably strengthen the host plant’s antioxidant defense system, significantly reducing the accumulation of ROS and malondialdehyde (MDA), thereby maintaining cellular integrity [5, 67]. Concurrently, mycorrhizal plants preserve higher photochemical efficiency of PSII, protecting the photosynthetic apparatus and sustaining carbon fixation under water deficit [5, 64]. Osmotic adjustment is another supported strategy, often observed as elevated accumulation of compatible solutes, such as proline. However, this relationship is nuanced. Increased drought tolerance occasionally occurs despite reduced proline accumulation. This underscores the importance of proline metabolism and turnover dynamics rather than simple accumulation [67, 68].

Furthermore, the symbiotic partnership relies on reciprocal nutrient exchange, wherein the host provides lipids to the fungus. Drought stress affects this lipid transfer. AMF enhance host drought tolerance by coordinated regulation of both symbiosis-specific and general fatty acid metabolism pathways. This highlights a critical biochemical interface underpinning drought response [69, 70].

PGPR constitute a diverse group of beneficial microorganisms capable of alleviating drought stress in tomato via numerous direct and indirect mechanisms (Table 1). The outcomes of interactions between PGPR and plants are significantly influenced by the host’s ET sensitivity, exemplifying genotype-dependent responses to biological regulators. For instance, *B. megaterium* reduces expression of ET biosynthesis genes in ET-sensitive wild-type tomatoes, conferring beneficial effects. In contrast, the same bacterial strain increases expression of ET-related genes in ET-insensitive *never ripe* mutants, thereby disrupting the beneficial interaction. This highlights that biostimulant efficacy depends on the genetic background of the cultivar [58, 59]. Other bacteria, including specific *Streptomyces* strains, can mitigate drought stress signaling. They transcriptionally suppress critical stress-responsive TFs such as *ERF1* [71].

**Table 1:** Summary of the effects of PGPR on the drought stress response in tomato.

**PGPR species**	**Isolation sources**	**Evidence level**	**Effects**	**Reference**
*Bacillus megaterium* and *Enterobacter* sp. *strain* C7	Southern Spain	Greenhouse	Enhanced the WUE of ET-sensitive tomato plants, *B. megaterium* and *Enterobacter* C7 PGPR activity in tomato plants under drought conditions might be dependent and independent on ET perception by ETR3, respectively	[58, 59]
*Streptomyces rimosus* C-2012 and *Streptomyces rochei* IT25	Iran	Greenhouse	Increased leaf relative water content (RWC), proline, MDA, hydrogen peroxide (H_2_O_2_) and total sugar content and APX activity and decreased CAT and GPX activity, inhibited expression of *ERF1* and *WRKY70*; alleviated drought stress	[71]
*Pseudomonas fluorescens* G20-18	Denmark	Greenhouse	Increased chlorophyll and ABA content, promoted stomatal closure, enhanced carbohydrate metabolic enzymes, antioxidant enzymes, and total antioxidant capacity, and strengthened the transcriptionally regulated systemic drought stress response	[72]
*Enterobacter* 64S1 and *Pseudomonas* 42P4	Argentina	Greenhouse	Increased growth parameters, total chlorophyll content, and chlorophyll fluorescence parameters of tomato plants under drought stress, increased endogenous MT and the accumulation of proline levels and reduced lipid peroxidation; improved tomato drought tolerance	[73]
*Diaporthe atlantica* EB4	Spain	Greenhouse	Improved photosynthetic capacity, nutrient uptake, enzymatic antioxidant response and osmo-protectant accumulation; enhanced drought tolerance	[74]
*Beauveria bassiana* D1–5	China	Laboratory	Enhanced the water absorption capacity and transpiration rate, increasing the density and size of stomata, peroxidase activity and proline content, reducing MDA; increased drought tolerance	[75]

PGPR also directly influence the host’s hormonal balance, thereby priming drought tolerance. The CTK-producing strain *Pseudomonas fluorescens* G20-18 elevates endogenous ABA concentrations in tomato, facilitating efficient stomatal closure. Moreover, bacterial CTKs function as cross-kingdom signaling molecules, triggering systemic responses characterized by enhanced chlorophyll content, elevated activity of antioxidant and carbon-metabolizing enzymes, and altered expression of drought-responsive genes [72]. Similarly, inoculation with MT-producing strains increases endogenous MT and proline concentrations, reduces lipid peroxidation of membranes, and enhances photosynthetic efficiency, collectively improving drought tolerance [73].

The list of effective PGPR includes other strains, such as the endophytic fungus *Diaporthe atlantica* EB4 [74], and the entomopathogenic fungus *Beauveria bassiana* D1-5 [75]. These organisms operate through multifaceted mechanisms, typically involving enhancement of photosynthetic performance, nutrient acquisition, antioxidant capacity, and osmoprotectant accumulation. Translating these promising laboratory and greenhouse findings into consistent large-scale field applications requires a deeper understanding of PGPR’s integrated effects on host morphology, physiology, and gene regulatory networks.

Various natural compounds derived from plants, algae, and other organic sources have shown substantial effectiveness in enhancing tomato drought tolerance, presenting sustainable tools for managing stress. Plant-derived protein hydrolysates, such as the biostimulant CycoFlow, have demonstrated improved performance in field conditions under water deficit. This improvement likely results from bioactive constituents, such as glycine betaine. These compounds influence physiological processes, including enhanced stomatal conductance and increased antioxidant compound concentrations. However, detailed molecular mechanisms require further study [76].

Algal extracts represent another significant category of biostimulants that improve drought tolerance in tomato. For example, the biostimulant ERANTHIS boosts antioxidant activity and significantly modulates gene expression. It affects genes linked to crucial functions such as AQP activity, TF regulation, and redox enzyme activities, thereby reducing cellular damage [77]. Extracts from *Ascophyllum nodosum* (ANE) also enhance drought tolerance, frequently by increasing levels of osmoprotectants and stress-related proteins. However, the efficacy of commercial ANE formulations varies considerably, emphasizing the importance of formulation and processing methods [78].

Furthermore, natural polymers derived from seafood waste, such as chitosan-chitin blends, offer eco-friendly alternatives for alleviating drought stress in tomatoes. Foliar application of these mixtures improves drought tolerance by enhancing essential physiological parameters, promoting stomatal closure, and modulating expression of stress-responsive genes such as *auxin response factor 4* (*SlARF4*), *SlWRKY81*, and *SlERF84* [79]. These diverse non-microbial biostimulants collectively demonstrate significant potential. They open avenues for developing innovative, environmentally sustainable strategies to enhance crop resilience under water-limited conditions.

In addition to macromolecular biostimulants, emerging evidence highlights the potential of small-molecule natural compounds for enhancing tomato drought tolerance through distinct mechanisms. β-Cyclocitral, a carotenoid-derived apocarotenoid, promotes a deeper and steeper root architecture by inducing meristematic cell divisions independently of auxin and BR signaling [80]. Among small secreted peptides, the C-terminally encoded peptide (CEP) family members SlCEP10 and SlCEP11b are drought-inducible and enhance drought tolerance by improving water status and reducing oxidative damage [81]. Conversely, the PSK peptide mediates drought-induced flower abscission. It is proteolytically activated by phytaspase 2 (SlPhyt2); silencing this protease reduces flower drop and improves fruit set [13]. Additionally, the natural antioxidant peptide PpT-2 mitigates drought-induced photosynthetic impairment by enhancing CO_2_ assimilation and PSII efficiency while decreasing oxidative stress [82]. These small-molecule compounds provide complementary mechanisms to traditional biostimulants. They represent a promising frontier for sustainable drought management in tomato.

### Non-biological regulators enhance tomato drought tolerance

Non-biological regulators offer a practical approach to improving crop resilience. Among these, MT has emerged as a multifunctional phytohormone essential for adaptation to abiotic stresses. Exogenous MT application, particularly at concentrations around 100 μM, consistently alleviates drought symptoms in tomato through multiple coordinated mechanisms (Fig. 3). It enhances root system architecture and vitality, maintains photosynthetic performance by protecting chloroplast integrity, and significantly strengthens antioxidant capacity by increasing enzymatic and non-enzymatic antioxidants [83]. At the molecular level, MT fine-tunes the hormonal balance. Notably, it suppresses the ABA biosynthesis gene *9-cis-epoxycarotenoid dioxygenase 3* (*SlNCED3*) and promotes the expression of genes involved in cuticular wax synthesis. This dual regulatory function reduces both stomatal and non-stomatal water losses [84, 85]. Under field conditions, lower-concentration MT foliar sprays (e.g. 20 ppm) effectively maintain growth during drought. They also preserve or enhance critical fruit quality traits, including soluble solids, ascorbic acid, and lycopene content [31].

**Figure 3:**
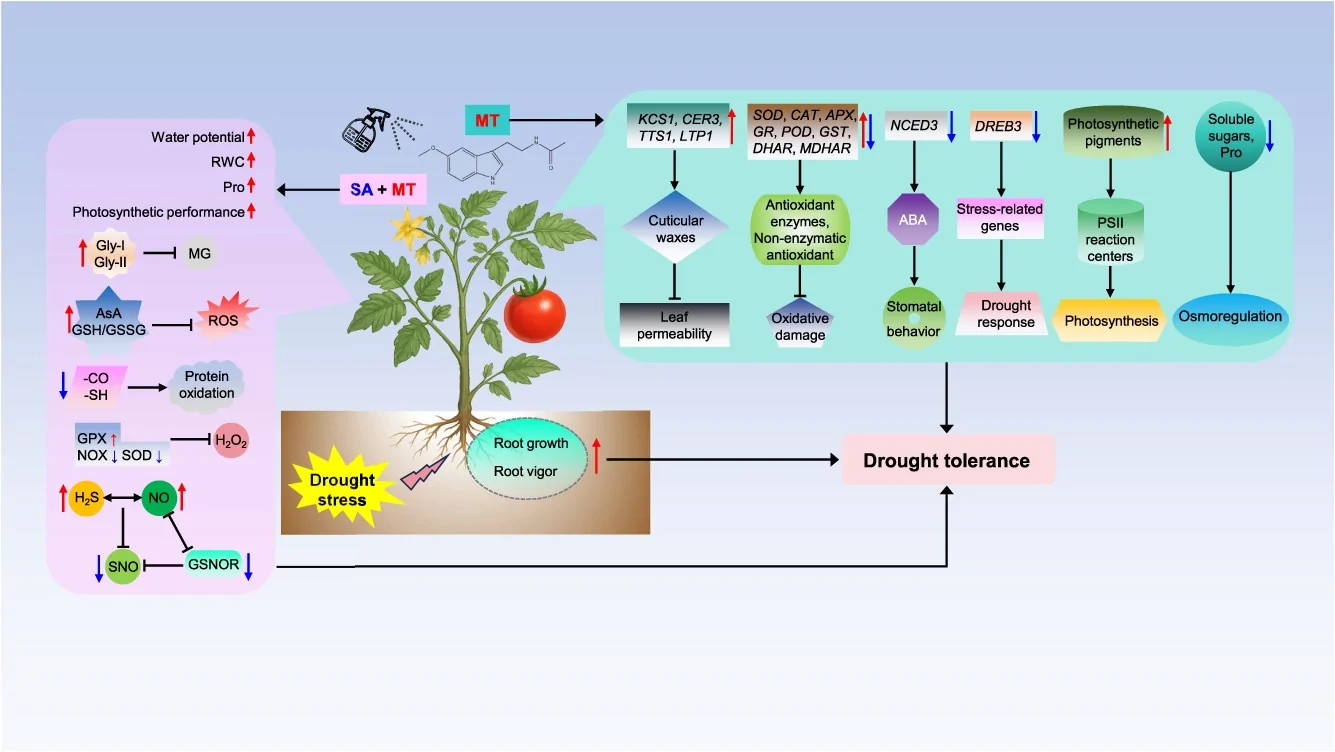
Mechanisms of MT and SA in regulating tomato drought tolerance. Exogenous MT enhances drought tolerance via multiple pathways: promoting root growth and leaf wax synthesis to reduce water loss; improving photosynthetic performance and antioxidant capacity; and modulating hormone levels (e.g. reducing ABA). On the other hand, MT and SA exhibit synergistic effects. Their combined application more effectively improves leaf water status, promotes the accumulation of osmoprotectants, and alleviates oxidative damage caused by drought stress through coordinated regulation of oxidative–nitrosative processes (e.g. enhancing the antioxidant system, modulating NO/H_2_S levels) and suppression of MG toxicity, thereby jointly enhancing tomato drought tolerance. Upward arrows and downward arrows represent increases and decreases in the corresponding indicators, respectively. RWC, relative water content; Pro, proline; -CO, carbonyl; -SH, thiol; GSSG, oxidized glutathione; GSNOR, S-nitroso glutathione reductase; MG, methylglyoxal; NOX, NADPH oxidase; NO, nitric oxide; GPX, glutathione peroxidase; Gly-I, Glyoxalase I; Gly-II, Glyoxalase II; SNO, S-nitrosothiol; SOD, superoxide dismutase; GSH, reduced glutathione.

Notably, MT does not function in isolation; it shares a biosynthetic precursor with SA, and their combined application demonstrates pronounced synergistic effects [84, 86]. Co-application of MT and SA surpasses individual treatments, yielding superior improvements in leaf water potential, relative water content (RWC), and osmotic adjustment via proline accumulation. Mechanistically, this synergy relies on coordinated enhancements in the oxidative–nitrosative system. Specifically, the combined treatment effectively scavenges ROS by optimizing the antioxidant redox state. It also modulates signaling molecules such as nitric oxide (NO) and hydrogen sulfide (H_2_S) by altering enzyme activities, including L-cysteine desulfhydrase (L-CD) and S-nitrosoglutathione reductase (GSNOR). Furthermore, it mitigates the toxicity of the reactive carbonyl methylglyoxal (MG) by upregulating the glyoxalase system [86]. The long-term agronomic potential and detailed signaling interactions of MT and SA warrant further research. However, the broad adoption of MT faces several practical challenges. Its cost-effectiveness compared to conventional agrochemicals remains unclear. Furthermore, regulatory frameworks for MT as an agricultural input are still developing in many regions. Additionally, its environmental fate requires thorough evaluation under realistic field conditions, particularly regarding impacts on nontarget soil microorganisms and aquatic ecosystems.

For silicon-nonaccumulating species such as tomato, silicon application is another effective strategy. Silicon alleviates drought primarily through its deposition in the apoplastic space, enhancing mechanical strength and minimizing water loss. Physiologically, silicon increases root hydraulic conductivity, improving water uptake from drying soils [87]. Additionally, it stimulates osmoprotectant accumulation and significantly reinforces the antioxidant defense system. This reduces membrane lipid peroxidation. Crucially, silicon maintains photosynthetic performance under stress. It prevents chlorophyll degradation, protects the PSII reaction center, and regulates photosynthesis-associated gene expression [88]. However, its efficacy is context-specific and decreases once drought intensity surpasses certain thresholds [87]. Similarly, selenium, another beneficial element, acts in a parallel manner. It enhances photosynthetic capacity and growth while reducing oxidative damage. An additional selenium-mediated mechanism involves increasing endogenous SA levels, creating a positive feedback loop in stress signaling [89]. Despite these advantages, applying beneficial elements poses certain challenges. Optimal application ranges for silicon and selenium are typically narrow, since excessive levels may induce phytotoxicity. Their effectiveness also depends on soil type, as bioavailability is influenced by soil pH and organic matter content. For selenium specifically, the risk of bioaccumulation necessitates careful management. Fruit concentrations must be maintained within safe limits for human consumption, which complicates its deployment in field conditions.

Apart from plant-targeted compounds, soil amendments such as biochar offer systemic benefits. The highly porous structure of biochar alters soil physical properties. It reduces bulk density and substantially enhances water retention capacity, thereby improving WUE under DI. Biochar, derived from organic wastes, also enhances soil fertility and NUE. Under drought stress, biochar application has been demonstrated to improve tomato growth, physiological status, final yield, and fruit nutritional quality. Notably, it increases lycopene content [90]. This enhancement results from improved soil moisture availability and direct or indirect physiological impacts on the plant [91, 92], potentially linked to altered ABA dynamics. Studies indicate that a biochar application rate of 0.2% (w/w) is more effective than 0.1%. This rate potentially enables significant irrigation volume reductions (~30%) without compromising yield or quality [90]. However, biochar efficacy declines when soil water content drops below 50% of field capacity. This highlights the need for complementary irrigation management. Ultimately, validation of these promising outcomes across diverse and complex open-field conditions is a critical next step toward broader adoption. Despite its benefits, biochar application faces several limitations. Its quality varies significantly depending on feedstock and pyrolysis conditions. Additionally, production, transportation, and soil incorporation entail substantial costs and energy investments. Long-term environmental risks, including potential alterations in heavy metal mobility and native soil carbon dynamics, require careful monitoring. Such economic and environmental factors are essential considerations for assessing the scalability and sustainability of biochar as a drought mitigation strategy in tomato production.

### Nanotechnology alleviates tomato drought stress

Recent development in nanotechnology have enabled the precise synthesis of nanomaterials. Due to their large surface-area-to-volume ratios and customizable properties, these nanomaterials provide innovative solutions for sustainable agriculture. These NPs can be absorbed by plants. They modulate physiological processes and enhance resilience to abiotic stresses, including drought. The effectiveness of NPs depends heavily on factors such as concentration, nanostructure, and application method. Both insufficient and excessive dosages often result in diminished or even negative effects.

Among the various studied NPs, zinc oxide (ZnO NPs) and silver NPs (Ag NPs) are extensively investigated for drought mitigation. Foliar application of these NPs, particularly in combination, reduces drought-induced damage in tomato plants. The primary mechanism involves enhancement of antioxidant enzyme activities, consequently lowering oxidative stress and associated lipid peroxidation. Additionally, such treatments elevate plant protein content. The combined application of ZnO and Ag NPs typically demonstrates greater efficacy compared to single-NP treatments [93]. Furthermore, ZnO NPs functionalized with compatible solutes such as proline and betaine not only mitigate oxidative stress but also enhance the concentrations of these osmolytes and lycopene in fruits, thus improving nutritional quality [94]. Notably, beneficial effects of ZnO NPs are concentration-dependent. Moderate concentrations (25–50 mg. L^−1^) effectively promote antioxidant activity and reduce oxidative markers, whereas higher doses may lead to phytotoxicity [95].

Beyond conventional metal-based NPs, other engineered nanomaterials have also demonstrated significant potential in improving tomato drought tolerance. These include titanium dioxide (TiO_2_), copper oxide (CuO), silica (SiO_2_), and chitosan NPs. Foliar-applied TiO_2_ NPs upregulate plasma membrane intrinsic proteins and photosynthesis-related proteins, thereby improving water retention and photosynthetic efficiency under drought conditions [96]. The effectiveness of SiO_2_ NPs is dependent on their structure. Porous and hollow structures enable faster release of silicic acid. This increases silicon uptake, enhances antioxidant enzyme activity, and facilitates coordinated regulation of auxin, JA, and SA signaling compared to solid or bulk forms [97]. Green-synthesized CuO NPs derived from plant extracts enhance drought tolerance by upregulating key genes in the phenylpropanoid pathway, thereby strengthening secondary metabolism and stress defense [98]. Chitosan NPs induce the expression of drought-responsive genes, including *heat stress TF A1a* (*HsfA1a*), *AREB1*, *NCED1*, and *plasma membrane intrinsic protein 1* (*PIP1*). These genes regulate heat shock responses, ABA signaling and biosynthesis, and AQP functions [99].

In addition to metal-based NPs, carbon-based and organic nanomaterials also exhibit efficacy. Nano-vermicompost and nano-biochar applications enhance photosynthetic performance and growth under drought by reducing oxidative stress and promoting accumulation of osmoprotectants and secondary metabolites [100, 101]. Functional carbon nanodots (FCNs) at optimal concentrations (e.g. 3 mg.kg^−1^) enhance photosynthesis and antioxidant activity while positively influencing soil microbial properties. Consistent with the dose-dependent patterns mentioned previously, higher concentrations of FCNs (10–30 mg.kg^−1^) may induce phytotoxicity [102]. Nanotechnology also facilitates stress monitoring. For instance, gold NP-based biosensors enable highly sensitive detection of drought-responsive miRNAs, providing valuable tools for assessing stress severity [103]. However, the precise molecular pathways underlying these benefits remain to be fully elucidated.

Despite their effectiveness under controlled conditions, translating these strategies into agricultural practice presents significant challenges. First, scalability and cost remain primary constraints. Green-synthesis methods typically have low yields, and large-scale production of uniform NPs is costly and energy-demanding. Second, environmental risks pose considerable concerns. The persistence and accumulation of metal-based NPs in soils are poorly understood. Comprehensive assessment of their long-term impacts on non-target organisms and their potential for bioaccumulation in food chains is required. Third, regulatory hurdles create a substantial bottleneck. A harmonized global regulatory framework for agricultural nanomaterials does not yet exist. Their classification as novel substances often requires extensive safety evaluations before commercial approval. Addressing these challenges requires interdisciplinary research, including the development of biodegradable nanomaterials and the establishment of clear environmental safety standards. This is essential for the widespread adoption for nanotechnology in field-scale drought management.

Beyond listing individual limitations, a critical comparative perspective is necessary to guide practical deployment. When compared with traditional non-biological regulators, such as MT and biochar, nanomaterials exhibit distinct trade-offs in cost-effectiveness and scalability. Traditional regulators benefit from more established application protocols and documented field efficacy. For instance, foliar MT at 20 ppm effectively maintained tomato growth, fruit yield, and quality attributes including total soluble solids, ascorbic acid, and lycopene under open-field drought conditions [31]. Biochar applied at 0.2% (w/w) significantly improved plant growth, fruit nutritional quality, and yield under drought stress. It also has the potential to save approximately 30% of irrigation water in greenhouse pot experiments [90]. These approaches are compatible with standard agricultural equipment and practices.

In contrast, the efficacy of nanomaterials has been demonstrated primarily in controlled greenhouse or growth chamber settings. Field-scale validation is largely lacking. Furthermore, their beneficial effects are highly concentration-dependent: ZnO NPs at 25–50 mg·L^−1^ enhanced antioxidant enzyme activities and biomass under drought, whereas 100 mg·L^−1^ induced phytotoxicity [95]. Similarly, carbon nanodots at 3 mg·kg^−1^ improved photosynthesis and yield, but higher doses (10–30 mg·kg^−1^) caused oxidative damage [102]. The efficacy of SiO_2_ NPs also depends critically on nanostructure. Porous and hollow forms outperform solid forms due to faster silicic acid release [97]. From an economic standpoint, MT is commercially synthesized as a pharmaceutical intermediate, and biochar can be produced from locally sourced organic wastes such as sugarcane bagasse [90], supporting cost-effectiveness and circular-economy compatibility. Green-synthesis methods for NPs offer environmental advantages [95, 98], but large-scale production with consistent particle characteristics and purity remains technically challenging.

Nevertheless, certain nanomaterials offer unique functionalities. TiO_2_ NPs up-regulate aquaporins and photosynthesis-related proteins to improve water retention and photosynthetic efficiency [96]. Gold NP-based biosensors enable ultrasensitive and specific real-time detection of drought-responsive miRNAs [103]. Traditional regulators cannot replicate these capabilities. In summary, for broad-acre field tomato production, traditional regulators such as MT and biochar currently provide more cost-effective and readily scalable solutions. For protected cultivation or precision agriculture systems, nanomaterials (particularly biodegradable and carbon-based formulations) represent a promising frontier, pending reductions in production costs and maturation of regulatory frameworks. Future research should prioritize comparative life-cycle cost analyses and multi-location field trials. These trials should directly benchmark representative nanomaterials against established regulator standards.

## Agronomic application strategies for tomato drought tolerance

Targeted agronomic management, comprising both cultivation practices and environmental controls, offers practical and scalable approaches to mitigate drought stress and secure tomato productivity. These strategies are founded on an understanding of tomato water relations, physiological responses, and soil–atmosphere interactions. This section systematically reviews essential strategies, evaluating their mechanisms, efficacy, and practical considerations.

### Cultivation strategies improve tomato drought tolerance

Grafting is a well-established horticultural practice originally developed against soil-borne pathogens. It has become a rapid and effective strategy for enhancing abiotic stress tolerance. By replacing the root system of a commercial scion with a resilient rootstock, grafting immediately improves root architecture and function. This approach avoids the lengthy process of breeding new varieties. Wild tomato relatives (e.g. *S. pennellii*) represent valuable genetic resources for rootstock breeding due to their superior root vigor and stress resilience [57]. For example, grafting onto interspecific hybrid rootstocks such as RF4A (*S. lycopersicum* × *S. pennellii*) enhances stomatal regulation and photosynthetic efficiency under water deficit. This improvement is primarily due to the robust, water-efficient root system inherited from the wild parent [57].

The performance of grafted plants depends strongly on the drought tolerance of the chosen rootstock. Compared to sensitive rootstocks, tolerant rootstocks substantially enhance scion growth under drought. They minimize oxidative damage and maintain superior photosynthetic capacity [20]. Importantly, successful grafting strategies aim to combine improved resilience with preserved yield and fruit quality. For instance, grafting onto the ‘Beaufort’ rootstock has been shown to improve drought tolerance without yield loss under moderate drought stress [104]. Beyond these intrinsic rootstock traits, long-distance signaling via the phloem plays a critical role in transmitting drought tolerance from rootstocks to scions. In tomato, the phloem-mobile cyclophilin SlCyp1 moves from wild-type scions to *dgt* mutant rootstocks. This movement restores auxin sensitivity, lateral root development, and xylem vessel formation. Consequently, it reduces the shoot-to-root ratio and improves water balance [105, 106]. This signaling function depends on SlCyp1’s enzymatic activity. It is enhanced under higher light intensity, linking shoot photosynthesis to root adaptive growth [106]. In addition, rootstock-to-scion transport of specific mRNAs contributes to drought tolerance in tomato. In tomato–wild tomato (*S. pennellii*) heterografts, hundreds of mobile mRNAs were identified under drought stress. These mRNAs were functionally enriched in photosynthesis and stress responses [107]. Small RNAs also act as long-distance signals. For example, in tomato–eggplant heterografts, rootstock-derived mobile sRNAs target scion genes involved in cell wall modification and stress signaling [108]. Collectively, rootstocks enhance scion drought tolerance in tomato through the delivery of mobile proteins, mRNAs, and sRNAs that reprogram gene expression and physiological responses. However, the widespread adoption of grafting remains limited due to higher seedlings costs and occasionally inconsistent performance under highly variable field conditions.

The application of organic compost serves dual purposes: enhancing soil fertility and increasing drought tolerance. Benefits arise from compost’s intrinsic physical and biological characteristics, such as high porosity, improved water retention capacity, and stimulation of beneficial microbial communities. These improvements create a more buffered rhizosphere environment. This slows moisture loss from soil and facilitates root function during drought. Research has indicated that certain composts, such as those derived from phosphate flotation waste and phosphogypsum, effectively mitigate drought-induced negative impacts on tomato growth, photosynthesis, and nutrient uptake [109]. Furthermore, combined application of different compost types can produce synergistic effects. This results in greater improvements in plant mineral content and WUE compared to single amendments [109]. Thus, integrating organic composts represents a sustainable soil management approach that supports tomato cultivation under water-limited conditions.

DI is a strategic water-saving practice. It involves irrigation at levels below the full crop evapotranspiration requirement. DI is based on the principle of enhancing WUE [110]. Although tomato yields generally correlate linearly with water availability, DI aims to apply irrigation selectively during less critical growth stages. This minimizes yield losses while achieving substantial water savings. However, DI success is context-dependent, as tomato sensitivity to water deficit varies with phenological stage. The reproductive stages, particularly flowering and fruit set, are notably vulnerable. Severe water deficits during these periods can cause significant yield reductions. Therefore, critical parameters include the timing, intensity, and duration of water deficit. Optimal DI regimes must be carefully calibrated. Factors to consider include local climate conditions, soil type, tomato cultivar, and integration with other water-conservation practices, such as mulching or grafting [110].

### Environmental control strategies alleviate drought stress in tomato

In controlled environment agriculture, managing aerial conditions directly influences plant water usage. eCO_2_ generally induces stomatal closure, reducing transpiration and enhancing WUE. In tomato, eCO_2_ exposure delays the reduction of photosynthetic rate and stomatal conductance during progressive soil drying. This helps maintain leaf area and biomass accumulation, and it improves nitrogen use efficiency (NUE) under drought [111]. This beneficial effect appears more pronounced under moderate or intermittent drought stress compared to severe, prolonged dehydration [112]. Interestingly, the mechanisms underlying stomatal closure may differ between ambient CO_2_ (aCO_2_) and eCO_2_ conditions. While ABA predominantly controls stomatal closure under aCO_2_, stomatal responses under eCO_2_ may involve alternative pathways, including SA signaling and hydraulic regulation [112, 113].

Crucially, altered stomatal behavior under eCO_2_ affects the effectiveness of established agronomic practices. DI depends on ABA-mediated stomatal sensitivity to soil drying. Under eCO_2_, stomatal sensitivity to ABA is reduced, and stomatal closure is delayed during gradual soil dehydration [113, 114]. This shift may alter the effective safety margin for DI. This margin is defined as the range in which stomatal closure limits water loss without significantly restricting carbon assimilation. Consequently, irrigation thresholds may need recalibration. To address this, we propose a shift from static, soil moisture–based DI to dynamic, plant-based irrigation strategies. These strategies are guided by real-time physiological indicators, such as leaf water potential [115] or plant electrophysiological signals [116]. These approaches offer direct feedback on stomatal activity under altered ABA sensitivity. Regarding VPD management, maintaining low VPD remains beneficial [117]; however, its relative importance may diminish as eCO_2_ itself restricts stomatal opening. Conversely, reduced ventilation may be used to sustain elevated CO_2_ concentrations. If this practice increases canopy temperature, the resulting rise in VPD could negate water-saving benefits. Thus, a coordinated CO_2_-VPD strategy, maintaining moderate eCO_2_ alongside optimal VPD, may synergistically conserve water [118, 119]. The precise interplay among CO_2_, VPD, and hormonal signaling in shaping drought responses warrants further investigation. Recent field trials have integrated DI with agrivoltaic systems. Regulated DI was based on plant water status and combined with photovoltaic panel shading. This approach reduced water consumption by 30%–45%, maintained fruit yield, and achieved land equivalent ratio values exceeded 1.5. These findings demonstrate the feasibility of dual land use for sustainable tomato production [120].

In summary, the effectiveness of agronomic strategies is not static. Under future eCO_2_ scenarios, changes in stomatal regulation necessitate recalibration of DI and VPD management. The most resilient systems will integrate real-time sensing and dynamic control. This will enable them to adapt to altered plant physiology and prevailing environmental conditions.

## Omics and design breeding strategies for tomato drought tolerance

Plant adaptation to drought stress involves integrated regulation of genes, proteins, and metabolites. Omics technologies (phenomics, genomics, transcriptomics, proteomics, and metabolomics) provide comprehensive, multi-dimensional insights into these complex responses at molecular, cellular, and organismal scales. Understanding the roles of drought-responsive components is essential for unraveling regulatory networks and accelerating genetic improvement. This section summarizes how these approaches facilitate the development of drought-tolerant tomato cultivars and highlights critical molecular candidates for targeted breeding.

### Omics approaches promote the breeding of drought-tolerant tomato varieties

Phenomics enables high-throughput quantification of physiological and morphological traits, bridging the gap between genotype and observable phenotype. It facilitates the selection of drought-tolerant genotypes by monitoring dynamic traits such as growth and WUE. For example, integrating sap ion dynamics with digital phenotyping has clarified physiological responses to drought [121]. Phenomic platforms can also efficiently identify optimal rootstock–scion combinations for improved water conservation [57]. However, the cost and complexity of phenotypic data acquisition remain significant barriers to broad adoption.

Genomics provides a fundamental basis for identifying genetic determinants of drought tolerance. The availability of a high-quality tomato reference genome has accelerated resequencing projects, enabling the discovery of genetic variations underlying stress adaptation. Studies involving drought-tolerant wild relatives (e.g. *S. pennellii*) have identified candidate genes contributing to enhanced drought tolerance [122]. Genome-wide association studies (GWASs) have further uncovered significant single-nucleotide polymorphisms (SNPs) linked to drought-tolerance traits. These SNPs offer valuable targets for marker-assisted selection (MAS) [123].

Transcriptomics has revealed the dynamic reprogramming of gene expression under stress conditions. Early microarray analysis identified numerous drought-responsive genes in tomato [124]. The advent of RNA sequencing (RNA-seq) has provided deeper and more comprehensive expression profiling. This approach has identified the coordinated suppression of genes related to photosynthesis and cell-cycle processes, alongside the induction of genes involved in ABA signaling, osmolyte synthesis, and stomatal regulation during drought stress and recovery phases [125].

Proteomics explores stress-induced translational and post-translational modifications, often revealing changes not fully captured at the transcript level. Comparative proteomic analyses between cultivated tomato and its drought-tolerant relative, *S. chilense*, have identified specific stress-responsive proteins, including hydrophilic and prefolding proteins activated by Ca^2+^ signaling [126]. Studies focusing on the chloroplast proteome have demonstrated significant drought-induced alterations persisting into the recovery stage, highlighting long-term impacts on the photosynthetic machinery [127]. Integrated transcriptomic–proteomic analyses confirm the importance of translational regulation in drought adaptation. However, interpretation of these datasets remains challenging.

Metabolomics characterizes the accumulation of primary and secondary metabolites associated with stress, offering a direct functional readout of plant physiological states. Studies have demonstrated that moderate drought conditions can improve fruit quality by increasing beneficial metabolites, including amino acids, antioxidants, and carotenoids [128]. Integration of metabolomics with other omics technologies is particularly powerful. For instance, combined phenomics and metabolomics have linked biostimulant-induced drought tolerance to specific metabolic shifts, such as altered secondary metabolism [129].

Before widespread adoption of omics technologies, forward-genetics approaches provided a critical foundation for dissecting the genetic basis of drought tolerance in tomato. In particular, quantitative trait locus (QTL) mapping using introgression line (IL) populations was instrumental. Using an IL population derived from *S. pennellii*, two major QTLs located on chromosomes 2 and 9 were identified. These QTLs regulate ~400 drought-responsive genes involved in transcriptional control, signaling, energy metabolism, and oxidative stress mitigation [124]. Subsequent studies using this genetic background identified five ILs exhibiting superior drought tolerance during reproductive stages. They also pinpointed candidate genes such as *sucrose non-fermenting-1-related protein kinase* (*SnRK*)*-Calcium-binding Sensor* (*SCS*), *L-cysteine desulfhydrase* (*LCD*), and *peroxiredoxin Q* (*PRXQ*) [130]. Similarly, from *S. pimpinellifolium*, four major QTLs controlling seed germination under drought stress were mapped to chromosomes 1, 8, 9, and 12 [131]. Using sub-near-isogenic lines from *S. habrochaites*, researchers mapped 19 QTLs associated with WUE and biomass under field conditions. Beneficial wild alleles conferred reduced C isotope discrimination (Δ^13^C) and increased shoot dry weight [132]. More recently, multi-parent advanced generation intercross (MAGIC) populations have enabled enhanced mapping resolution. This allowed fine-mapping of 24 drought-related QTLs to intervals smaller than 2 Mb. It also facilitated candidate gene prioritization through integration with transcriptomic data [133, 134]. Collectively, these forward-genetic studies have identified stable QTL regions and favorable alleles from wild relatives. Integration with transcriptomic and metabolomic datasets connects these QTLs to specific candidate genes underlying drought tolerance. These genomic resources thus provide valuable targets for marker-assisted breeding and functional gene discovery.

These integrative approaches demonstrate that multi-omics strategies can significantly advance a systems-level understanding of drought responses. In tomato, weighted gene co-expression network analysis (WGCNA) has been utilized to integrate transcriptomic datasets from drought-treated wild and cultivated varieties. This approach enables identification of hub genes and key metabolic pathways coordinately regulated under stress [135]. Similarly, combining multiple omics layers has facilitated the prioritization of candidate genes within mapped QTL intervals. For instance, integrating QTL mapping and transcriptomic analysis in tomato recombinant inbred line populations revealed stable QTLs for seedling drought tolerance. This identified candidate genes such as *methionine sulfoxide reductase 1* (*SlMSRB1*) and *BR insensitive 1-like receptor* (*SlBRL1*) [136]. More recently, a metabolite-based genome-wide association study (mGWAS) integrated with transcriptomics uncovered two biosynthetic gene clusters and their positive regulator R2R3 MYB TF SlMYB13. This regulator controls phenolamide accumulation and consequently enhances drought tolerance in tomato [137]. Such integrative approaches not only refine candidate gene lists but also elucidate regulatory networks underlying drought adaptation. However, effectively synthesizing these diverse datasets to model the complex physiological and biochemical changes associated with drought tolerance remains a significant challenge. Addressing this issue depends on continued development of user-friendly computational pipelines and standardized data-sharing platforms. Progress in these areas will ultimately facilitate the identification of molecular markers and target genes that reliably influence complex agronomic traits. Translating these integrative findings into breeder-friendly tools will be essential for simultaneously improving drought tolerance and marketable yield.

Looking forward, integrating multi-omics data with artificial intelligence (AI)–assisted phenomics and predictive modeling will accelerate breeding for drought-tolerant tomato cultivars. High-throughput phenotyping combined with machine learning (ML) enables rapid selection of superior genotypes under field conditions [138]. Predictive models integrating genomic, transcriptomic, and metabolomic data can forecast genotype performance under drought scenarios [139]. To maximize translational impact, omics-derived targets should be incorporated into breeder-accessible strategies rather than relying exclusively on transgenic approaches. Candidate genes and QTLs can be converted into diagnostic markers for MAS, enabling introgression of beneficial alleles from wild relatives. Genomic selection (GS) has demonstrated feasibility for traits such as fruit weight and soluble solids content in tomato. It shows considerable promise for accelerating genetic improvement for abiotic stress tolerance, including drought [140]. Complementarily, CRISPR-based genome editing enables precise modification of validated negative regulators or stacking beneficial alleles. Collectively, MAS, GS, and genome editing form a robust translational pipeline. This pipeline leverages omics discoveries to expedite the development of drought-tolerant tomato varieties directly applicable to breeding programs.

### Genetic engineering strategies for tomato drought tolerance

Advances in functional genomics have identified numerous candidate genes suitable for genetic engineering aimed at improving drought tolerance in tomato (Table 2). Drought tolerance mechanisms in tomato involve intricate, multi-layered gene networks that regulate critical biological processes, including signal perception, transcriptional regulation, physiological metabolism, and stress responses. This section systematically reviews the roles of these genes and their associated regulatory pathways (Fig. 4), aiming to provide a theoretical basis for the genetic enhancement of drought tolerance in tomato.

**Table 2:** Summary of genes involved in drought stress in tomato.

**Gene name**	**Category/type**	**Evidence level**	**Function**	**Phenotype under drought stress**	**Reference**
*OPEN STOMATA 1* (*SlOST1*)-*VASCULAR PLANT ONE-ZINC FINGER 1* (*SlVOZ1*)	Protein kinase (SlOST1); NAC-type transcription factor (TF) (SlVOZ1)	Laboratory	SlOST1–SlVOZ1 module regulated the flowering-integrator gene *SINGLE FLOWER TRUSS* (*SFT*), thereby balancing drought response and reproductive development	Promoted flowering transition	[10]
*Demethylase alpha-ketoglutarate-dependent dioxygenase B (AlkB) homolog 9B (SlALKBH9B)*	Demethylase	Laboratory, greenhouse	Suppressed drought-induced flower drop by reducing ethylene production via enhancing *ethylene excess producer 1* (*SlETO1*) mRNA stability and translation	Negatively regulated drought-induced flower drop	[11]
*Like heterochromatin protein 1b (SlLHP1b)*	Histone H3K27me3-binding protein	Laboratory, greenhouse	Bound histone H3 in the lysine 27 (H3K27me3) to epigenetically silence flavin monooxygenase *SlYUC* and inhibit auxin synthesis	Promoted drought-induced flower drop	[12]
*SlMYB92 and ALIPHATIC SUBERIN FERULOYL TRANSFERASE (SlASFT)*	MYB TF (SlMYB92); feruloyl transferase (SlASFT)	Laboratory	Knockout of *SlMYB92* significantly reduced exodermal suberin deposition; mutation of *SlASFT* reduced suberin monomers and disrupted lamellae	Enhanced drought tolerance	[15]
*Hydrotropism-associated loci (SlMIZ1-1)*	DUF617 domain-containing protein	Laboratory	Essential for tomato root hydrotropism, drought-induced expression, and drought recovery	Enhanced drought tolerance	[16]
*DOWNY MILDEW RESISTANCE 6-1 (SlDMR6-1)*	2-oxoglutarate (2OG) and Fe (II)-dependent oxygenase	Laboratory, greenhouse	Mutation of *SlDMR6-1* increased SA levels, reduced transpiration and stomatal conductance, up-regulated antioxidant genes, down-regulated ABA catabolism gene *SlCYP707A2*	Negatively regulated drought tolerance	[19]
*ABA-responsive element binding protein 1 (SlAREB1)*	bZIP TF	Laboratory	Up-regulated stress-related genes, mediated ABA-dependent drought stress responses	Enhanced drought tolerance	[26]
*Solanum pennellii DnaJ1(SpDnaJ1)*	Heat shock protein	Laboratory	Reduced ROS accumulation, increased antioxidant activity, and interacted with flavanone 3-hydroxylase-like protein (F3HL) to promote F3H production	Enhanced drought tolerance	[29]
*Rho of Plants 9 (SlROP9)*	Rho GTPase	Laboratory, greenhouse, field	Null mutants of *SlROP9* reduced stomatal conductance and transpiration without yield penalty, via respiratory burst oxidase homolog (RBOH)-dependent ROS accumulation in guard cells	Negatively regulated water use efficiency (WUE)	[30]
*Calcium-dependent protein kinase 27* (*SlCPK27*)	Calcium-dependent protein kinase	Laboratory	Directly interacted with and phosphorylated tonoplast sugar transporter 2 (TST2), promoting intercellular soluble sugar accumulation	Enhanced drought tolerance	[34]
*Polyamine oxidase 3 (SlPAO3)*	Polyamine oxidase	Laboratory	Mutation of *SlPAO3* increased thermospermine, reduced xylem hydraulic conductivity, and promoted stem elongation	Negatively regulated drought tolerance	[37]
*Spermidine synthase 1 and 4 (SlSPDS1/4)*	Spermidine synthase	Laboratory, greenhouse	Increased specific polyamines/tyramines, reduced ROS/chlorophyll loss	Enhance drought tolerance	[38]
*Indole-3-acetic acid 9(SlIAA9)*	IAA protein	Laboratory, greenhouse	Induced by stress and hormones, suppressed ROS scavenging	Negatively regulated drought tolerance	[44]
*Ethylene-responsive factor D2 (SlERF.D2)*	ERF TF	Laboratory	Degraded via ABA-CPK27-Plant U-box 22 (PUB22) to derepress jasmonic acid (JA) synthesis	Negatively controlled drought tolerance	[45]
*Mitogen-activated protein kinase 3* (*SlMAPK3*)	Mitogen-activated protein kinase	Laboratory	Increased photosynthetic activity, chlorophyll content, proline and sugar levels, alleviated oxidative stress	Enhance drought tolerance	[48, 49]
*MYB 78 like (SlMYB78-like)*	R2R3-MYB TF	Laboratory	Represses the ABA degradation gene *SlCYP707-A2*, interacted with ABA-related factor SlDREB3, enhanced ABA accumulation	Enhance drought tolerance	[50]
*ABA-responsive element binding factor2 (SlABF2)*	bZIP TF	Laboratory	Activated sly-miR398b to repress Cu/Zn superoxide dismutase 1 (SlCSD1), promoted superoxide-induced stomatal closure, interacted with SnRK2.6	Enhance drought tolerance	[51]
*Small secreted peptide 10* (*SlCEP10*); *small secreted peptide 11b* (*SlCEP11b*)	Small secreted peptides	Laboratory	Improved water status, reduced oxidative damage	Enhance drought tolerance	[81]
*MYB TF 13* (*SlMYB13*)	MYB TF	Greenhouse	Promoted phenolamide accumulation, induced the scavenging of ROS and the increase of ABA content	Enhanced drought tolerance	[137]
*CLAVATA3/EMBRYO SURROUNDING REGION-related 10 (SlCLE10)*	CLE peptide	Laboratory, greenhouse	Promoted root hair growth and drought tolerance via the SIMAPK6-SlACS2-ethylene module, with cross-species activity	Enhanced drought tolerance	[142]
*Annexin-interacting universal stress protein 2* (*SlAnnSp2*)	Annexin	Laboratory, greenhouse	Regulated ABA synthesis, scavenged ROS	Enhanced drought tolerance	[143]
*SlOST1-Slow anion channel associated 1–6 (SlSLAC1–6)*	Protein kinase (SlOST1); slow anion channel (SlSLAC1–6)	Laboratory	SlOST1-SlSLAC1 protein module promoted stomatal closure, reduced oxidative damage, and maintained chloroplast structure	Enhanced drought tolerance	[144]
*BRASSINOSTEROID-INSENSITIVE 1 (SlBRI1)*	BR receptor	Laboratory	Increased BR signaling intensity, slightly improved contents of ABA and ROS, reduced both stomatal aperture and drought tolerance	Negatively regulated drought tolerance	[145]
*Alternative oxidase 1a* (*SlAOX1a*)	Mitochondrial alternative oxidase	Laboratory	Reduced H_2_O_2_ accumulation and promoted autophagy	Positively regulated drought tolerance	[146]
*Glucose-6-phosphate dehydrogenase* (*SlG6PDH*)	Glucose-6-phosphate dehydrogenase	Greenhouse, field	Induced by drought, increased reductant supply, and was linked to ABA signaling, proline synthesis, Hsp70, and ascorbate peroxidase	Positively regulated drought tolerance	[147]
*Uridine diphosphate glycosyltransferase 52 (SlUDPGT52)*	Uridine diphosphate glycosyltransferase	Laboratory, greenhouse	Knockout of *SlUDPGT52* improved ROS scavenging	Negatively regulated drought tolerance	[148]
*Non-repressible 5-related N-acetyltransferase 2 (SlSNAT2)*	Chloroplast-localized acetyltransferase	Laboratory	Catalyzed the Lys acetylation of Rubisco lysine; *slsnat2* mutant accumulated endogenous ABA, enhanced the activity of antioxidant enzymes, improved photosynthesis	Negatively regulated drought tolerance	[149]
*WRKY TF 8 (SlWRKY8)*	WRKY TF	Laboratory	Promoted the accumulation of substances, enhanced the expression of stress response genes, reduced stomatal aperture, increased the activity of antioxidant enzymes	Enhanced drought tolerance	[150]
*Nonexpressor of pathogenesis-related gene 1 (SlNPR1)*	Master regulator involved in plant defense response to pathogens	Laboratory	*slnpr1* mutant increased stomatal aperture, higher electrolytic leakage, MDA and H_2_O_2_ levels, and lower activity levels of antioxidant and expression levels of drought related key genes	Positively regulated drought tolerance	[151]
*Respiratory burst oxidase homolog B (SlRbohB)*	Nicotinamide adenine dinucleotide phosphate (NADPH) oxidase	Laboratory	Silencing *SlRbohB* accelerated water loss, altered expression of drought-responsive genes	Positively regulated drought tolerance	[152]
*Plasma membrane intrinsic protein 2;1, 2;7, 2;5 (SlPIP2;1, SlPIP2;7, SlPIP2;5)*	Aquaporin (AQP) proteins	Laboratory	Improved plant water content and maintained osmotic balance, promoted root water uptake	Enhanced drought tolerance	[153]
*Δ^1^-pyrroline-5-carboxylate reductase (SlP5CR)*	Δ^1^-pyrroline-5-carboxylate reductase	Greenhouse	The variant mimicking S-nitrosylation (*SlP5CR^C5W^*) promoted growth, increase P5CR activity, proline content, and WUE, reduced ROS enhanced drought tolerance	Enhanced drought tolerance	[154]
*MicroRNA 159 (SlmiR159)*	microRNA	Laboratory	Targeted SlMYB33, promoted the accumulation of the osmotic protection compounds proline and putrescine	Enhanced drought tolerance	[155]
*JUNGBRUNNEN1 (SlJUB1)*	NAC (NAM, ATAF and CUC) TF	Laboratory	Inhibiting *SlJUB1* increased ion leakage and H_2_O_2_ levels, decreased the expression of various drought-responsive genes	Enhanced drought tolerance	[156]
*NAC TF 11 (SlNAC11)*	NAC TF	Greenhouse	Induced by dehydration, cold, and heat; positively regulated ABA sensitivity	Positively regulated drought tolerance	[157]
*Basic helix–loop–helix TF 22 (SlbHLH22)*	Basic helix–loop–helix TF	Greenhouse	Improved ROS scavenging system, increased osmotic potential and enhanced accumulation of secondary metabolites	Improved drought tolerance	[158]
*Basic helix–loop–helix TF 96 (SlbHLH96)*	Basic helix–loop–helix TF	Laboratory	Stimulated the expression of genes encoding antioxidants, ABA signaling molecules and stress-related proteins, physically interacted with SlERF4, repressed *SlCYP707A2* expression	Improved drought tolerance	[159]
*WRKY TF 6 (SlWRKY6)*	WRKY TF	Laboratory	Enhanced antioxidant capacity and activated ABA synthesis/signaling	Enhanced drought tolerance	[160]
*WRKY TF 81 (SlWRKY81)*	WRKY TF	Laboratory	Suppressed proline biosynthesis and nitric oxide-mediated stomatal closure, reduced photosynthetic capacity and increased water loss	Negatively regulated drought tolerance	[161, 162]
*Lycopene ε-cyclase gene (SlLCY-E) allele*	Lycopene ε-Cyclase	Greenhouse	*SlLCY-E* allele (G/3378/T) increased β,β-xanthophyll in leaves and boosted fruit lycopene/carotenoids, elevated ABA levels	Enhanced drought tolerance	[163]
*Late embryogenesis abundant protein 6 (SlLEA6)*	Late embryogenesis abundant proteins	Greenhouse	Silencing *SlLEA6* impaired antioxidant and ROS defense systems, increased cellular damage	Positively regulated drought tolerance	[164]
*PHYTOCHROME B1 (SlPHYB1)*	Photoreceptor phytochrome	Greenhouse	Positively regulated fruit yield during reproductive-phase drought, and negatively regulated fruit soluble solids	Negatively regulated vegetative growth	[165]
*Uridine diphosphate glucosyltransferase 75C1 (SlUGT75C1)*	ABA uridine diphosphate glucosyltransferase	Greenhouse	Glycosylated ABA and IAA, negatively regulated ABA levels, delayed fruit ripening, promoted seed germination	Reduced drought tolerance	[166]
*Natural allele of B-box zinc finger protein 18 (SlBBX18^TT^)*	Truncated B-box zinc finger TF	Greenhouse, field	Repressed *ascorbate peroxidase 1* (*APX1*) expression via direct promoter binding; the natural C-terminal truncation (*SlBBX18^TT^*) increased drought sensitivity	Negatively regulated drought tolerance	[167]
*Basic leucine zipper TF 1 (SlbZIP1)*	Basic leucine zipper TF	Greenhouse	Modulated ABA pathway and stress-related gene expression	Positively regulated drought tolerance	[168]
*Cold-inducible GT factor (ShCIGT)*	Trihelix TF	Laboratory, greenhouse	Interacts with SnRK1, reduced sensitivity to ABA	Enhanced drought tolerance	[169]
*Ethylene response factor B1 (SlERF.B1)*	AP2/ERF TF	Laboratory	Inhibited the expression of stress-responsive genes	Increased sensitivity to drought stress	[170]
*Heat-shock TF A1a (HsfA1a)*	Heat-shock TF	Laboratory	Activated autophagy-related (ATG) genes and induced autophagy	Induced drought tolerance	[171]
*MYB TF 50 (SlMYB50)*	R2R3-MYB TF	Laboratory	Silencing *SlMYB50* reduced ROS, improved physiological parameters, and upregulated stress-related genes	Negatively regulated drought tolerance	[172]
*Plant AT-rich sequence and zinc-binding 17 (SlPLATZ17)*	Zinc finger TF	Laboratory	Interacted with POR1 in response to stress, might regulate the accumulation of ROS	Increased drought tolerance	[173]
*Solanum lycopersicum BES1 3 (SlSLB3)*	BRI1-EMS-SUPPRESSOR 1 (BES1) TF	Laboratory	Silencing *SlSLB3* increased ROS and MDA, decreased SOD, and altered BR-related gene expression	Positively regulated drought tolerance	[174]
*Zinc-finger TF 31 (SlZF-31)*	C2H2 TF	Laboratory	Silencing *SlZF-31* reduced the contents of ABA, SOD, POD and proline, increased the MDA content, lowered the chlorophyll fluorescence parameters	Positively regulated drought tolerance	[175]
*LATERAL ORGAN BOUNDARIES DOMAIN 40 (SlLBD40)*	LBD TF	Laboratory	Induced by PEG, salt, and methyl jasmonate; knockout of *SlLBD40* enhanced drought resistance	Negatively regulated drought tolerance	[176]
*Auxin response factor 4 (SlARF4)*	ARF TF	Laboratory	Knockout of *SlABF4* enhanced drought tolerance via ABA signaling and morphological changes	Negatively regulated drought tolerance	[177]
*AGD2-like defense response protein 1 (SlALD1)*	Aminotransferase involved in pipecolic acid (Pip) biosynthesis	Laboratory	Knockout of *SlALD1* enhanced drought tolerance by improving photosynthesis and antioxidant capacity	Negatively regulated drought tolerance	[178]
*WUSCHEL-related homeobox 4 (SlWOX4)*	WOX TF	Laboratory	Knockout of *SlWOX4* enhanced drought tolerance by promoting stomatal closure, reducing ROS, and increasing antioxidant activity and ABA content	Negatively regulated drought tolerance	[179]
*Trihelix TF (SlGT30)*	Trihelix TF	Laboratory, greenhouse	Knockout of *SlGT30* enhanced drought tolerance and fruit size	Negatively regulated drought tolerance	[180]
*WRKY TF 16 (SlWRKY16)*	WRKY TF	Laboratory, greenhouse	Physically interacted with CONSTANS Interacting Protein 2b (CIP2b), bound to the promoter of syntaxin gene *SlSYP121* and repressed its expression	Negatively regulated drought tolerance	[181]

**Figure 4:**
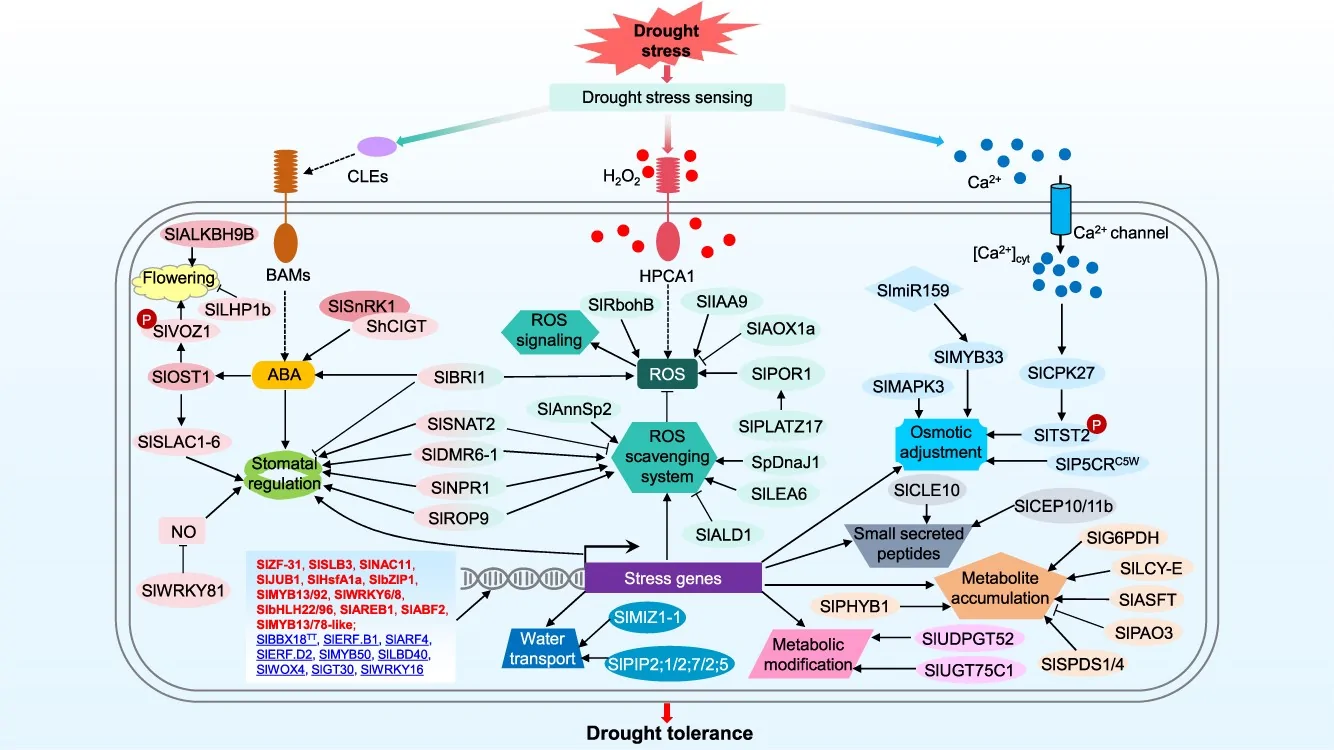
Regulatory network of tomato response to drought stress. The core mechanisms include: ABA signal transduction and stomatal regulation, encompassing a regulatory network centered on the protein kinase SlOST1, which orchestrates flowering and stomatal closure, as well as positive and negative regulators modulating stomatal movement through the ABA signaling pathway; ROS homeostasis and antioxidant defense, comprising ROS signaling and ROS scavenging systems that alleviate oxidative damage; osmotic adjustment and metabolite accumulation, involving the accumulation of proline, soluble sugars, and other metabolites to maintain water balance; transcriptional regulation, including the regulation of downstream stress-responsive gene expression by various TFs such as NAC, WRKY, bHLH, and MYB, thereby affecting the physiological response of tomato plants to drought stress; and other physiological and biochemical processes, including water transport, metabolic modification, and small peptide signaling. These pathways synergistically enhance the drought adaptability of tomato. Solid arrows indicate promotion, dashed arrows indicate putative promotion, and T-shaped arrows indicate inhibition. Fonts in bold represent TFs that positively regulate drought tolerance, while underlined fonts represent TFs that negatively regulate drought tolerance. P, phosphorylation; NO, nitric oxide; [Ca^2+^]_cyt_, cytosolic calcium ions; CLEs, CLAVATA3/Embryo surrounding region peptides; BAMs, BARELY ANY MERISTEM receptor kinases; HPCA1, H_2_O_2_-induced Ca^2+^ increases 1; SlBBX18^TT^, C-terminally truncated SlBBX18 protein; SlP5CR^C5W^, a S-nitrosylation-mimicking variant of SlP5CR.

Hormonal signaling and biosynthesis genes represent primary targets in drought response. In *Arabidopsis*, the CLAVATA3/EMBRYO-SURROUNDING REGION-RELATED25 (CLE25)-BARELY ANY MERISTEM (BAM) module mediates long-distance transmission of root dehydration signals to leaves, thereby promoting ABA accumulation and enhancing drought tolerance [141]. Whether a functionally analogous regulatory pathway operates in tomato remains to be fully elucidated. Under drought stress, SlCLE10 in tomato roots activates the mitogen-activated protein kinase 6 (SlMAPK6). SlMAPK6 subsequently phosphorylates SlACS2, which enhances the stability and catalytic activity of SlACS2 and thereby stimulates ET production. The resulting changes substantially increase the length and density of tomato root hairs, effectively strengthening plant adaptation to drought stress. Exogenous application of low-dose SlCLE10 peptide significantly improves drought tolerance in diverse crop species, including Solanaceae, Brassicaceae, and Cucurbitaceae. The evolutionary conservation of SlCLE10-mediated drought tolerance across plant taxa highlights its potential as a promising target for genetic improvement of crop stress tolerance [142]. The annexin gene *SlAnnSp2* enhances drought tolerance by promoting ABA biosynthesis and ROS scavenging [143]. The kinase SlOST1 mediates stomatal closure through phosphorylation of slow anion channel associated 1–6 (SlSLAC1–6) and influences drought-induced flowering by stabilizing SlVOZ1 [10, 144]. In contrast, *BR-INSENSITIVE 1* (*SlBRI1*) negatively influences drought tolerance despite increasing ABA content. This reveals antagonistic interactions between BR and ABA signaling pathways [145]. However, the precise molecular mechanisms underlying these interactions remain incompletely characterized.

Under drought stress, H_2_O_2_ generated in plant cells is recognized by H_2_O_2_-induced Ca^2+^ increases 1 (HPCA1) [182]. The signal is further transduced to regulate plant stress responses. Nevertheless, excessive ROS cause oxidative damage to biological macromolecules and may even result in plant cell death. Antioxidant defense and ROS homeostasis genes are crucial in managing oxidative stress. *Alternative oxidase 1a* (*SlAOX1a*) regulates mitochondrial ROS, contributing to ET-mediated drought tolerance and autophagy [146]. Knockout of *uridine diphosphate glycosyltransferase 52* (*SlUDPGT52*) via CRISPR/Cas9 enhances drought tolerance by improving ROS scavenging capacity [148]. Additionally, mutation of *nonrepressible 5-related N-acetyltransferase 2* (*SlSNAT2*) promotes tolerance through increased ABA accumulation and elevated antioxidant enzyme activities [149]. This underscores the tight connection between ROS homeostasis and hormonal regulation. Nevertheless, most studies have focused on single genes, lacking a comprehensive analysis of dynamic equilibrium within the ROS metabolic network.

Stomatal and water transport regulatory genes directly modulate water loss. *SlWRKY8* helps maintain leaf water content by reducing stomatal aperture [150]. In contrast, mutation of *nonexpressor of pathogenesis-related gene 1* (*SlNPR1*) has the opposite effect, decreasing tolerance [151]. Moreover, silencing of *respiratory burst oxidase homolog B* (*SlRbohB*) accelerates water loss and alters the expression of drought-responsive genes [152]. AQP genes such as *SlPIP2;1*, *SlPIP2;7*, and *SlPIP2;5* are vital for root water uptake and osmotic balance [153]. Collectively, these studies indicate coordinated multi-gene regulation of water homeostasis under drought stress. However, the stability and consistency of these responses across diverse genetic backgrounds and environmental conditions require further validation.

Osmotic adjustment genes facilitate osmolyte accumulation. Tomato *Δ1-pyrroline-5-carboxylate reductase* (*SlP5CR*) enhances drought tolerance by increasing proline biosynthesis, improving WUE, and reducing ROS accumulation [154]. SlCPK27 promotes soluble sugar accumulation through phosphorylation of the sugar transporter SlTST2 [34]. Additionally, the microRNA miR159 increases proline and putrescine concentrations by targeting *SlMYB33* [155]. However, the metabolic flux reorganization associated with osmotic adjustments may consume substantial carbon and nitrogen resources. This could potentially compromise tomato fruit yield and quality. The balance and overall impact of these metabolic changes under field conditions remain unclear.

TFs serve as master regulators within stress-responsive networks. NAC family TFs such as *JUNGBRUNNEN1* (*SlJUB1*) and *SlNAC11* act as positive regulators of drought tolerance [156, 157]. bHLH TFs, including *SlbHLH22* and *SlbHLH96*, enhance drought tolerance through ROS scavenging, ABA signaling, and osmotic adjustment [158, 159]. Similarly, WRKY TFs like *SlWRKY6* and *SlWRKY8* improve drought tolerance via ABA-mediated stomatal control and antioxidant defense [150, 160]. In contrast, *SlWRKY81* may negatively regulate drought responses by suppressing NO accumulation and proline synthesis [161, 162]. These TFs frequently function within modular regulatory networks, as exemplified by the SlbHLH96-SlERF4 module [159]. Nevertheless, upstream and downstream regulatory pathways, as well as crop-specific regulatory networks, require further clarification.

Post-translational modification genes serve as rapid and precise regulatory switches under drought stress. SlSNAT2 acetylates Rubisco, influencing photosynthesis and drought tolerance [149]. Phosphorylation of SlVOZ1 by SlOST1 constitutes a crucial step for ABA-dependent nuclear translocation [10]. These findings demonstrate that post-translational modifications function as molecular switches, facilitating rapid adaptation to drought. However, dynamic monitoring and engineering applications of these modifications remain technically challenging.

Compared to conventional transgenic approaches, genome editing technologies, particularly CRISPR/Cas9, enable precise modification of target genes. They provide powerful tools to dissect gene function and directly enhance drought tolerance. In tomato, targeted mutagenesis of negative regulatory genes has proven effective. For instance, knockout of *LATERAL ORGAN BOUNDARIES DOMAIN 40* (*SlLBD40*) enhances drought tolerance by reducing water loss, alleviating membrane damage, and improving stomatal regulation [176]. Similarly, disruption of *SlARF4* improves drought tolerance through modulation of stomatal closure and reduced ROS accumulation [177]. Knockout of *AGD2-like defense response protein 1* (*SlALD1*) also confers improved drought tolerance via activation of photosynthetic and antioxidant systems [178]. In addition, loss-of-function mutations in *WUSCHEL-related homeobox 4* (*SlWOX4*) promote ABA signaling and stomatal closure [179]. In contrast, knockout of *SlMAPK3* reduces drought tolerance, indicating its role as a positive regulator [49]. Moreover, *de novo* domestication of stress-tolerant wild species by editing key domestication genes provides a promising approach. This can rapidly generate tolerant germplasm [183, 184]. These studies illustrate that genome editing represents a highly efficient and precise platform for developing drought-tolerant tomato cultivars. Future efforts will likely emphasize multiplex and base-editing approaches to pyramid beneficial traits.

Beyond knockouts, recent studies have identified novel gene targets where loss of function enhances drought tolerance. Some of these targets also confer benefits for fruit yield. For example, CRISPR/Cas9-mediated knockout of the trihelix TF *SlGT30* reduces stomatal density and promotes endoreduplication. This leads to enhanced drought tolerance and increased fruit size and weight, thereby breaking the typical trade-off between stress tolerance and productivity [180]. Similarly, a natural SNP in *SlWRKY16*, which causes premature translation termination, results in a truncated protein that relieves repression of the syntaxin gene (*SlSYP121*). The SlWRKY16-CONSTANS Interacting Protein 2b (CIP2b)-*SlSYP121* module regulates ROS homeostasis and stomatal closure. Genome editing of this module offers a strategy to enhance drought tolerance [181].

When pyramiding favorable drought-tolerance alleles from wild relatives using CRISPR/Cas9, a primary challenge is balancing stress resilience with productivity. To minimize unintended impacts on yield and quality, we propose a three-pronged framework. First, precision editing techniques such as base editing or promoter editing enable subtle adjustments of gene activity without complete loss-of-function. For example, base editing–mediated correction of a deleterious mutation in the *SUPPRESSOR OF SP2* (*SSP2*) gene improves tomato architecture and early yield [185]. Second, tissue-specific or stress-inducible CRISPR systems can limit gene modifications to relevant tissues (e.g. roots or guard cells), thus avoiding interference with reproductive and fruit developmental processes. This approach is informed by genes such as *SlWRKY75*, which exhibit divergent roles in roots compared to shoots [186]. Several tissue-specific promoters have been experimentally validated in tomato and could serve as candidates for such systems. For root-targeted expression, the tomato *root-expressed 2-ODD* (*SlREO*) promoter drives strong, dehydration-insensitive expression. Its activity is specifically confined to the root cortex of mature tomato plants [187]. For guard-cell-specific expression, the potato voltage–dependent potassium ion channel (*KST1*) promoter has been used to drive *Arabidopsis ABF4* expression in transgenic tomato, with expression predominantly in guard cells. Transgenic lines showed significantly improved drought tolerance without the severe growth retardation often caused by constitutive expression of stress-related TFs [188]. Third, parallel assessments of yield components (fruit number, size) and quality parameters (soluble solids, lycopene) under both well-watered and drought conditions enable early detection of pleiotropic effects. Collectively, these strategies offer a roadmap for effectively harnessing wild allelic diversity while safeguarding commercial yield and fruit quality.

Beyond conventional CRISPR/Cas9 knockout strategies, newer precision editing tools enable fine-tuning of drought-responsive genes without inducing double-strand breaks (DSBs). Base editors (cytosine base editor CBE and adenine base editor ABE) convert C•G to T•A or A•T to G•C base pairs without DSBs or donor templates [189]. Prime editing (PE) uses a Cas9 nickase-reverse transcriptase fusion guided by a prime editing guide RNA (pegRNA). This system directly copies desired edits into target DNA, enabling all 12 base substitutions and small indels without DSBs [189]. In tomato, an ultra-efficient PE (UtPE) system was developed by integrating evolved PE6 variants, an altered pegRNA, an RNA chaperone, and a geminiviral replicon. This system achieved 3.39–8.89-fold higher efficiency than PE2max and up to 87.5% desired edits in *T*_0_ plants, with stable inheritance and low off-target effects [190]. Furthermore, precise knockin of a 10-bp heat-shock element into cell-wall invertase promoters via PE conferred heat-responsive upregulation. This enhanced carbon partitioning and increased yield by 33% in tomato under heat stress [191]. Although that study focused on heat stress, the same strategy can be applied to drought tolerance by inserting DRE or ABRE motifs into promoters of key drought-related genes. These DSB-free technologies complement CRISPR/Cas9 knockout. They are particularly valuable for modulating drought-tolerance genes where complete loss of function may incur yield penalties. These technologies, while still evolving, complement CRISPR/Cas9 mutagenesis and are poised to accelerate the development of drought-tolerant tomato varieties with minimal unintended consequences.

Omics technologies have greatly accelerated the identification of drought tolerance mechanisms in tomato. They reveal intricate networks involving genes, proteins, and metabolites. Genetic engineering, guided by these discoveries, provides a powerful means for precise trait modification. The future of breeding will depend on effectively integrating multi-omics datasets to engineer resilient, high-yielding tomato cultivars. It is also crucial to ensure the stability of these engineered traits across diverse environments.

## Smart technologies for climate-resilient production systems

Recent advances in digital twin (DT), ML, and Internet of Things (IoT) technologies have facilitated the development of climate-resilient irrigation systems. These systems integrate real-time environmental sensing, predictive modeling, and automated control.

### Digital twin–based irrigation management

A DT creates a virtual model of a physical agricultural system synchronized in real time, enabling monitoring, prediction, and control [192]. In processing tomato, the IrriDesK DT was validated over two growing seasons under semi-arid conditions. It integrated soil moisture sensors, weather data, and seasonal water allocation thresholds [115]. Regulated DI guided by the DT reduced water consumption by 30%–45% compared to full irrigation. It maintained yields of 90–108 t·ha^−1^ and increased fruit soluble solids by 10%–15%, while leaf water potential and normalized difference vegetation index confirmed adequate crop water status [115].

### Machine learning–based decision support

A 3-year field trial evaluated an ML-based decision support system (DSS). The system combined a Random Forest model for soil water balance prediction with a genetic algorithm for irrigation optimization [193]. Under DI (80% ETc), the system achieved water savings of 32.66% without significant yield reduction. During a year of severe thermal stress, water productivity remained comparable to full irrigation, demonstrating the system’s capability to sustain productivity under combined water and heat stresses [193].

### Edge Internet of Things and reinforcement learning for real-time control

An edge IoT architecture using LoRa communication provided coverage exceeding 10 000 m and facilitated distributed machine-to-machine communication [194]. Irrigation scheduling employed the Optimal Soil Wetness Closeness Policy (OSWCP). This policy is a reinforcement learning algorithm (RL-PPO) integrated with Penman–Monteith evapotranspiration modeling. Field validation over 56 days demonstrated 97.88% accuracy in irrigation recommendations and achieved water savings of 55% compared to manual irrigation while preserving soil health [194].

### Artificial intelligence–driven plant electrophysiological monitoring

A noninvasive electrophysiological (EP) sensor integrated with a convolutional neural network (CNN) enabled direct, real-time monitoring of plant water status [116]. The system achieved a rapid 29-second response from data acquisition to valve actuation. It detected deviations of 10% from optimal irrigation levels and reduced water use by 20% per cycle while maintaining optimal plant hydration [116].

Collectively, these studies demonstrate that systems based on DT, ML, IoT, and AI can significantly reduce water use (20%–55%) while maintaining or enhancing tomato yield and quality under drought conditions. Future research should prioritize integration with predictive climate models, extending sensor technologies to additional crops, and developing scalable, cost-effective solutions suitable for smallholder farmers.

## Integrated strategies for enhancing tomato drought tolerance

Integrating complementary approaches consistently outperforms single strategies. These approaches include sequential combinations of early stress priming with late-stage management, synergy between genotype selection and optimized nutrient-water management, pairing DI with soil amendments, co-application of microbial consortia with organic or industrial by-products, and the combined use of nano-fertilizers and hydrogels. Such integration achieves synergistic effects rather than merely additive ones, with emergent benefits derived from functional complementarity and mechanistic interactions among component strategies.

### Temporal integration of stress acclimation and abiotic regulation

Early mild stress conditions prime plants, enhancing their tolerance to subsequent abiotic stresses. Seedling drought hardening followed by low-salinity irrigation (2 g·L^−1^) during the reproductive stage reduced soil salt accumulation, alleviated root water–uptake inhibition, and activated cross-stress defense memory [195, 196]. Mild hardening optimized root architecture; promoted selective sodium uptake and leaf excretion; and maximized yield, fruit quality, and WUE under combined drought and salt stress [197]. This temporal approach enables sequential application of interventions, such as MT treatment or grafting, tailored to specific phenological stages.

### Genotype screening with nutrient–water management

Targeted integration of genotype-specific drought adaptability with optimized nutrient–water management enables synergistic improvements in yield and drought tolerance. For example, combining variety selection based on sensitivity to water stress with precise regulation of the C/N ratio increased total phenolic content in ‘Pebroter’ tomatoes without yield reduction. In contrast, the ‘Montserrat’ variety showed no such response [198]. Additionally, integrating landrace varieties with organic amendments under DI at 70% irrigation effectively mitigated water stress. Additive main effects and multiplicative interaction modeling demonstrated distinct varietal differences in yield and WUE responses [199]. Thus, effective nutrient–water management relies heavily on matching interventions with the genetic adaptability of the selected genotype.

### Deficit irrigation combined with soil improvement

Moderate DI (75% field capacity) enhances water productivity while maintaining yield and soil microbial stability [200]. Under such conditions, AMF inoculation increased red fruit yield by ~25%, improved root water uptake and nutrient acquisition, and boosted soil microbial biomass [201]. In commercial greenhouse trials, combining AMF inoculation with sensor-driven DI and reduced fertilization reduced water use by 20% and increased water productivity by 10%–14%. This approach achieved value–cost ratios ranging from 6 to 12 [202].

### Multicomponent synergy of microbial agents with organic or industrial by-products

Integrating functional microbial consortia with organic amendments or industrial by-products achieves multidimensional regulation of the soil–plant–microbe system. A six-strain PGPR consortium under drought conditions (40% field capacity) improved RWC, antioxidant enzyme activities, expression of stress-responsive genes, soil properties, and increased yield by 51.09% [203]. AMF–compost combinations functionally complemented each other, significantly elevating antioxidant compound levels in tomato fruits under severe drought (35% field capacity) [204]. Phosphogypsum (PG) alone inhibited tomato growth. However, combining PG with PGPR and AMF alleviated its toxicity. This was achieved through microbial siderophore production, phosphorus solubilization, and exopolysaccharide secretion, simultaneously promoting waste valorization and drought tolerance [205].

### Water–fertilizer–soil–microbe integration via nano-fertilizers and hydrogels

In arid regions, combining nano-fertilizers and hydrogels forms an integrated four-in-one synergistic approach. Applying nano-diammonium phosphate (Nano-DAP) together with hydrogels improved soil water-holding capacity up to 78%–79%, a threefold increase. This combination also decreased bulk density and enhanced soil organic carbon, nutrient availability, and microbial biomass [206]. The hydrogel component optimized soil water status and structure, while Nano-DAP facilitated nutrient release and microbial activity. This combined strategy significantly enhanced tomato plant growth and yield, surpassing the efficacy of single-component applications [206].

To provide operational guidance for implementing the G × E × M framework under field conditions, we developed a decision matrix (Table 3) and a stepwise flowchart (Fig. 5) based on the evidence synthesized in this review. These tools are designed to help practitioners, including farmers, extension agents, and breeders, select context-specific strategies according to local soil texture, drought severity, climatic conditions, and available tomato varieties.

**Table 3:** Decision matrix for selecting drought-tolerance strategies based on local G × E × M conditions.

**Local condition (E)**	**Genotype availability (G)**	**Recommended management strategies (M)**	**References**
Sandy soil, low water retention	Sensitive commercial variety (e.g. ‘Moneymaker’)	Biochar (0.2% w/w) + DI (75% field capacity)	[90, 200]
Clay loam, moderate drought	Moderately tolerant landrace (e.g. ‘Ciettaicale’)	AMF inoculation + reduced irrigation (20%–30% water saving)	[6, 14, 63, 201]
Arid region, high VPD	Any scion variety	Grafting onto drought-tolerant rootstock (e.g. ‘Beaufort’, RF4A) + eCO_2_ enrichment (if controlled environment)	[57, 104, 111]
Saline-affected area, combined stress	ET-sensitive variety	PGPR (e.g. *B. megaterium*) + low-salinity irrigation (2 g·L^−1^) after seedling hardening	[58, 59, 195, 196]
High temperature + drought	Widely adapted variety	MT (100 μM or 20 ppm foliar) + AMF	[4, 5, 31]
Low-fertility soil, limited inputs	Local landrace	Organic compost (phosphate flotation waste-based) + DI (70% ETc)	[109, 199]
Protected cultivation (greenhouse)	High-yielding commercial hybrid	Sensor-driven DI + structurally optimized TiO_2_ or SiO_2_ NPs	[96, 97, 115]

**Abbreviations:** VPD, vapor pressure deficit; ET, ethylene; DI, deficit irrigation; ETc, crop evapotranspiration; AMF, arbuscular mycorrhizal fungi; PGPR, plant growth–promoting rhizobacteria; MT, melatonin; NPs, nanoparticles.

**Figure 5:**
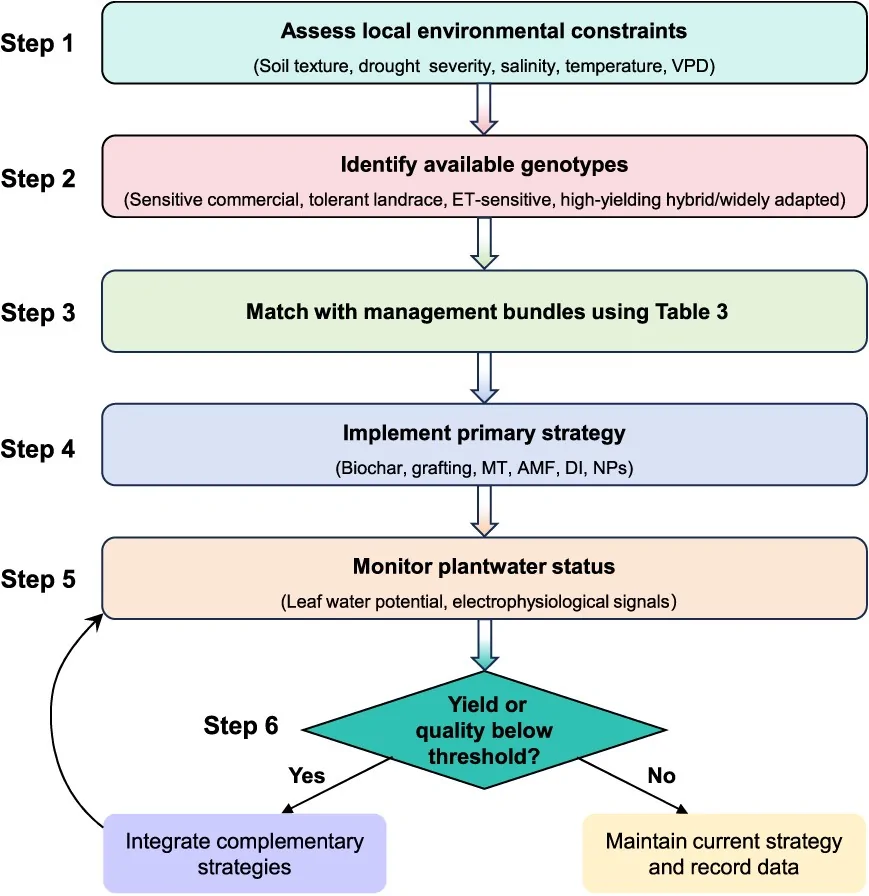
Operational G × E × M decision flowchart for enhancing drought tolerance in tomato. The flowchart guides practitioners from environmental assessment (Step 1) through genotype identification (Step 2), strategy matching (Step 3, based on Table 3), primary implementation (Step 4), real-time monitoring (Step 5, including indicators such as leaf water potential or electrophysiological signals), and adaptive integration of complementary strategies if yield or quality falls below a threshold (Step 6). Arrows indicate the decision pathway. VPD, vapor pressure deficit; ET, ethylene; MT, melatonin; AMF, arbuscular mycorrhizal fungi; DI, deficit irrigation; NPs, nanoparticles.

This operational framework directly translates the descriptive G × E × M principles into actionable steps. It emphasizes that no single strategy is universally applicable. Instead, successful drought mitigation in tomato requires matching genotype, environment, and management based on locally available resources and constraints. Future research should prioritize field validation of these decision matrices across multiple locations. Furthermore, user-friendly digital tools (e.g. mobile apps) that integrate local weather and soil data should be developed to deliver real-time recommendations.

The practical significance of this integrated perspective lies in its real-world applicability. Farmers and practitioners can combine locally available resources with precision management approaches to develop resilient tomato production systems. Future research should prioritize field-scale validation of these integrated strategies. It should also focus on developing decision-support tools for optimizing multifactor combinations and elucidating the molecular mechanisms underlying the observed synergistic interactions.

## Challenges and bottlenecks in field translation

Despite numerous strategies validated under controlled conditions, translating these results into consistent field applications remains challenging. The inherent complexity of open-field and commercial greenhouse systems often leads to inconsistent outcomes. A critical bottleneck is the limited availability of strategies that reliably demonstrate efficacy across diverse real-world environments. Some methods, such as grafting onto resilient rootstocks (e.g. ‘Beaufort’ or interspecific hybrids like RF4A), consistently preserve yield under moderate drought [57, 104]. However, the effectiveness of other approaches varies significantly. For example, the efficacy of AMF inoculation is limited by soil phosphorus levels. High phosphorus conditions suppress symbiosis through hormonal interactions, particularly via GA-mediated pathways [207]. Similarly, commercial biostimulants like ANEs show considerable variability in efficacy due to differences in formulation, processing methods, and source materials [78]. For nanomaterials such as TiO_2_ and SiO_2_ NPs, benefits have been documented under controlled conditions [96, 97]. Nevertheless, open-field performance remains variable and highly sensitive to factors including application timing, dosage, and NP structural characteristics.

The adoption of novel strategies is further shaped by economic, environmental, and management trade-offs. Economically, the high cost of grafted seedlings, precision irrigation systems, and specialized biostimulants can be prohibitive, particularly for smallholder systems. In addition, the cost-effectiveness of MT application remains unclear. Environmentally, the use of metal-based nanomaterials raises concerns regarding their long-term fate, accumulation, and effects on nontarget organisms. Similarly, beneficial elements such as selenium pose risks of bioaccumulation in fruits if not carefully managed. Variability in biostimulant formulations and the absence of standardized quality control further reduce consistency. From a management perspective, many strategies require a level of precision and technical expertise that may not be readily available. For instance, mistimed DI during the sensitive flowering stage can lead to severe yield loss. Similarly, silicon and selenium have narrow optimal concentration ranges; beyond these ranges, phytotoxicity may occur. In addition, microbial consortia require specific handling conditions to maintain viability.

Ultimately, successful field implementation is constrained by interactions among soil type, climate, and genotype × environment (G × E) factors. A strategy effective in one soil context may not perform similarly in another. For example, the water-retention benefits of biochar decline when soil moisture falls below 50% of field capacity [90]. Likewise, eCO_2_ alleviates drought stress more effectively under moderate than under severe conditions [112]. Genotype selection plays a critical role in determining outcomes. The same PGPR strain may exert contrasting effects depending on host ET sensitivity [58, 59]. Moreover, a drought-tolerant rootstock that performs well under controlled conditions may behave differently in field environments due to variations in climate, disease pressure, or soil microbiota. Therefore, a robust G × E × M framework is essential to tailor strategies to local conditions. Otherwise, the gap between laboratory findings and field performance will persist.

## Challenges and future perspectives

Despite substantial progress in elucidating and enhancing tomato drought tolerance, translating laboratory findings into reliable field applications remains a major challenge. Controlled experimental environments often fail to capture the complexity of field conditions, resulting in unstable performance of promising genes, biostimulants, or nanomaterials. A key limitation is the intrinsic trade-off between stress tolerance and productivity. Central pathways such as ABA signaling regulate both drought responses and normal growth, meaning genetic modifications may incur unintended penalties, including reduced yield. Furthermore, the high carbon cost associated with osmolyte biosynthesis may compete with fruit development. Moreover, the genetic mechanisms underlying the coexistence of high yield and drought tolerance remains insufficiently understood.

This trade-off operates at multiple levels. First, drought redirects carbon from reproductive sinks to defensive functions. In the sensitive cultivar ‘Moneymaker’, stress-induced sugar accumulation reflects disrupted source–sink relationships rather than enhanced tolerance. In contrast, the tolerant landrace ‘Ciettaicale’ maintains PSII efficiency and root starch reserves while increasing its root-to-shoot ratio [6, 14]. Second, drought-induced hormonal changes impose a growth penalty. Water deficiency suppresses bioactive GA levels via downregulation of biosynthesis genes and upregulation of the deactivating gene, leading to DELLA-mediated leaf growth inhibition [18]. Concurrently, ABA induces the IAA oxidase gene *SlDAO2* through the SlAREB1/SlABI3-1/SlABI5 transcriptional cascade, promoting local auxin deactivation and inhibiting hypocotyl elongation [42]. This exemplifies a mechanism by which stress signaling can directly antagonize growth-promoting hormonal pathways. Collectively, carbon redirection toward protective sinks, GA-mediated growth restriction, and ABA-driven auxin antagonism impose an ‘opportunity cost’ on fruit development.

In addition to these biological constraints, the effective integration of rapidly expanding multi-omics datasets presents another major challenge. The generation of multi-omics data has outpaced our capacity for integrative analysis, limiting the development of predictive models linking genotypes to drought-related phenotypes. This issue is further compounded by the ‘black-box’ nature of deep learning models, which reduces interpretability and limits their adoption by breeders [208]. Moreover, the interactions among multiple factors, including rhizosphere microbiota, soil amendments, irrigation regimes, and microclimatic conditions, remain poorly characterized. These context-dependent interactions, combined with environmental variability across sites, complicate the design of optimized integrative strategies.

To overcome these challenges, future research should adopt a systems-level framework integrating complementary technologies. Dimensionality reduction approaches, such as non-negative matrix factorization (NMF), can transform high-dimensional molecular data into representative meta-features, enabling efficient GWASs across multiple omics layers [209]. Advanced deep learning models, including autoencoders and convolutional neural networks (CNNs), can denoise and extract latent features from transcriptomic and metabolomic datasets, facilitating reconstruction of regulatory networks governing stress responses [208, 210]. ML approaches enable cross-layer data integration, improving trait prediction and identification of key gene targets. AI-driven tools such as DeepCRISPR further optimize guide RNA design and off-target prediction, supporting the generation of genome-edited lines with minimal unintended effects [211]. In parallel, CRISPR/Cas9 genome editing enables precise pyramiding of beneficial alleles, including those derived from wild tomato relatives, thereby enhancing drought tolerance while minimizing associated trade-offs.

However, the commercial deployment of genetically engineered drought-tolerant tomatoes faces significant regulatory and public acceptance hurdles. Regulatory frameworks diverge globally. The European Union applies strict Genetically Modified Organism (GMO) regulations to most genetically engineered crops [212]. The USA adopts a product-based trigger for regulation [213]. China follows a middle-ground approach [214]. Public acceptance remains a critical bottleneck. Consumers generally favor transgene-free genome-edited products over traditional transgenic GMOs, yet knowledge of both technologies remains low [215]. CRISPR-edited tomatoes face considerable consumer resistance. Substantial benefits, such as a significant reduction in pesticide use, are required to gain acceptance [216]. In Japan, the first CRISPR-edited tomato reached the market, yet consumer concerns over labeling persist [217]. To bridge this translational gap, we recommend prioritizing transgene-free Site-directed Nuclease 1 (SDN-1) edits, transparent labeling [217], early stakeholder engagement, and support for harmonized international guidelines [215].

Beyond these socio-regulatory considerations, genetic advancements must be integrated with tailored agronomic practices within a structured G × E × M framework. A practical implementation of this framework would involve: (i) genotype characterization using AI-enhanced genomic prediction models that incorporate agroclimatic variables to simulate germplasm performance in target environments; (ii) environmental monitoring utilizing unmanned aerial vehicles (UAVs) equipped with multispectral and thermal sensors, along with IoT-based soil sensors; (iii) management optimization through ML models trained on multi-location field trials to predict yield and WUE under various irrigation strategies; and (iv) integration via multimodal deep-learning frameworks that fuse genotypic, phenotypic, and environmental data to predict breeding values across diverse environments. Fundamental research should also enhance understanding of key interface processes, including root–microbe chemical interactions and integrated stomatal regulation under future climate scenarios (e.g. eCO_2_). Such studies can be facilitated by AI-driven analyses of root architecture and time-series imaging.

Ultimately, the effectiveness of these strategies must be evaluated by their simultaneous enhancement of field yield and fruit quality. Developing practical assessment systems to quantify trade-offs among WUE, yield, and quality is essential. This approach will shift the research emphasis from isolated ‘drought tolerance’ toward the integrated goal of ‘water-saving, yield-stable, and high-quality’ tomato production.

## Conclusions

This review systematically evaluates integrated approaches to enhance drought tolerance in tomato, including molecular mechanisms, omics-assisted breeding, regulator applications, agronomic management, and nanotechnology. Central challenges remain in bridging laboratory and field conditions, balancing drought tolerance with fruit yield and quality, and achieving deeper interdisciplinary integration.

Looking forward, we propose three priority research directions. First, integrating multi-omics datasets with AI-assisted predictive modeling should transform high-dimensional data into interpretable models. These models can then accurately forecast genotype performance across diverse field environments. This strategy should be supported by explainable AI and user-friendly analytical pipelines. Second, a structured G × E × M optimization framework is required. This framework should systematically evaluate genotype–management combinations across target environments. It should leverage IoT sensing, UAV-based phenomics, and ML. Third, mechanistic dissection of critical regulatory nodes should prioritize pathways with high translational potential. These include the ABA signaling hub (SlOST1-SlVOZ1), hormonal crosstalk networks governing stomatal control and flower abscission, and regulatory modules controlling root architecture and ROS homeostasis.

Among the most promising breeding targets, we highlight three key areas. First, SlOST1 and its downstream effectors integrate drought sensing with flowering and stomatal regulation. Second, root architecture traits from wild relatives enhance water uptake with minimal yield penalties. Third, ROS homeostasis modules sustain photosynthetic efficiency and fruit quality under stress. CRISPR-based genome editing enables the pyramiding of favorable alleles. Genomic selection that incorporates multi-omics data provides a pathway to accelerate genetic improvement.

## Data Availability

No data was used for the research described in the article.
